# The Role of AI in Improving Digital Wellness Among Older Adults: Comparative Bibliometric Analysis

**DOI:** 10.2196/71248

**Published:** 2026-01-14

**Authors:** Naveh Eskinazi, Moti Zwilling, Adilson Marques, Riki Tesler

**Affiliations:** 1 Economics and Business Administration Department, Ariel University, Ramat Hagolan 65 Street, Ariel, 40700, Israel, 972 0543007323; 2 Faculty of Human Kinetics, University of Lisbon, Lisbon, Portugal; 3 Health Management Department, Ariel University, Ariel, Israel

**Keywords:** digital wellness, artificial intelligence, digital divide, digital inclusion, mHealth, mobile health, older people

## Abstract

**Background:**

Advances in artificial intelligence (AI) have revolutionized digital wellness by providing innovative solutions for health, social connectivity, and overall well-being. Despite these advancements, the older population often struggles with barriers such as accessibility, digital literacy, and infrastructure limitations, leaving them at risk of digital exclusion. These challenges underscore the critical need for tailored AI-driven interventions to bridge the digital divide and enhance the inclusion of older adults in the digital ecosystem.

**Objective:**

This study presents a comparative bibliometric analysis of research on the role of AI in promoting digital wellness, with a particular emphasis on the older population in comparison to the general population. The analysis addressed five key research topics: (1) the evolution of AI’s impact on digital wellness over time for both the older and general population, (2) patterns of collaboration globally, (3) leading institutions’ contribution to AI-focused research, (4) prominent journals in the field, and (5) emerging themes and trends in AI-related research.

**Methods:**

Data were collected from the Web of Science between 2016 and 2025, totaling 3429 documents (344 related to older people), analyzed using bibliometric tools.

**Results:**

Results indicate that AI-related digital wellness research for the general population has experienced exponential growth since 2016, with significant contributions from the United States, the United Kingdom, and China. In contrast, research on older people has seen slower growth, with more localized collaboration networks and a steady increase in citations. Key research topics for the general population include digital health, machine learning, and telemedicine, whereas studies on older people focus on dementia, mobile health, and risk management.

**Conclusions:**

The results of our analysis highlight an increasing body of research focused on AI-driven solutions intended to improve the digital wellness among older people and identify future research directions to refer to the specific needs of this population segment.

## Introduction

Information technology, the internet, and artificial intelligence (AI) have emerged as transformative domains that shape contemporary life [1]. Technology-driven alternatives have increasingly replaced traditional services, revolutionized daily routines, and fostered connectivity and convenience for a growing global population. Despite these advancements, the older population often encounters significant challenges adapting to these technological changes. These include barriers related to accessibility, digital literacy, and the complexity of operating modern devices and affiliated services [2].

Digital literacy is essential for older populations, particularly as essential services increasingly transition online; yet, many older adults face significant barriers in adopting these technologies. Research indicates that anxiety related to using information and communication technologies can hinder engagement, leading to frustration and helplessness, which further exacerbates the digital divide [3]. A recent scoping review highlighted that older adults often exhibit low digital health (DH) literacy, with many lacking the necessary skills to navigate DH resources effectively [4]. Additionally, studies have revealed that limited access to technology and inadequate infrastructure contribute to the exclusion of older adults from digital life, particularly in rural areas [5]. Furthermore, intergenerational support has been shown to positively influence digital participation among older people in rural settings, suggesting that fostering such support could enhance digital literacy outcomes [6]. Overall, targeted educational interventions are crucial to improve digital skills and reduce anxiety, promoting greater inclusion and engagement with digital resources among older adults [7].

Recent research by Anisha et al [8] has demonstrated the overall positive attitudes of older adults toward DH technology acceptance, with studies showing that the technology acceptance model (TAM) and the unified theory of acceptance and use of technology are the most frequently used frameworks for evaluating technology acceptance among this population. Key facilitators of technology acceptance include perceived usefulness, ease of use, social influence, and digital or eHealth literacy, while barriers involve technical challenges, usability issues, and privacy concerns. However, the acceptance of AI-based conversational agents for noncommunicable disease management among older adults remains inadequately evaluated, possibly due to limited adaptation of established frameworks to specific health care contexts and technology innovations.

Studies have shown that customized interventions are crucial for successful technology acceptance among the older population, with core components of TAM including perceived usefulness, perceived ease of use, attitude toward use, behavioral intention to use, subjective norms, image, and facilitating conditions [9]. Challenges arising from TAM applications in older people’s health care include technological literacy barriers, digital divide concerns, privacy and security apprehensions, resistance to change, limited awareness and information, health conditions and cognitive impairment, trust and reliability concerns, a lack of tailored interventions, overcoming age stereotypes, and integration with traditional health care.

Meta-analytic evidence conducted by Yang et al [10] has revealed significant positive correlations between perceived usefulness, perceived ease of use, and social influence with behavioral intention to use health care technology among older adults, with moderating effects based on geographic region, technology type, and presence of visual demonstrations. These findings suggest that tailored strategies for different types of technology and the use of visual demonstrations are important for enhancing adoption rates among older adults.

These challenges are further intensified by the growing dependency on digital tools in key areas of interaction for older individuals, including health care services, legal systems, social connections, information access, and the integration of interconnected smart devices within the home environment [11,12].

The advent of AI has introduced a range of transformative tools that offer innovative solutions tailored to the unique needs of older individuals. For instance, AI-powered personalized health care services have demonstrated the potential to bridge the gap between technological advancements and the older people’s orientation to digital tools, significantly enhancing their quality of life [13,14]. These technologies not only address accessibility challenges but also create pathways for older people to engage meaningfully with digital ecosystems such as health care [15]. Research on middle-aged adults’ acceptance of AI chatbots has shown moderate acceptance levels, with perceived ease of use, subjective norm, and user image significantly influencing users’ intention to use AI chatbots, highlighting the importance of preparing for aging with personalized technology [16].

The rapidly expanding integration of AI across various sectors has been documented through comprehensive bibliometric analyses, which reveal significant growth patterns and emerging trends. Educational technology research has shown a notable rise in AI-related studies beginning in 2018, with citations reaching their zenith in 2019, and collaborative metrics indicating that the United States and China are leading in publication volume [17]. Similarly, research on AI in education has rapidly progressed, with studies demonstrating that China, the United States, India, Spain, and Germany lead in research productivity, with particular emphasis on higher education compared to K-12 education [18]. The intersection of AI and language learning has also gained substantial attention, with bibliometric analysis revealing a rising pattern of AI applications in language learning processes, identifying influential authors, institutions, and countries contributing to this growing field [19]. Furthermore, the convergence of AI with environmental, social, and governance frameworks has emerged as an evolving research area, with increasing publications indicating the growing importance of sustainable AI applications [20]. The integration of AI into learning management systems has also demonstrated significant potential, offering adaptive and personalized learning experiences that promote active learning and support self-regulated learning across face-to-face, hybrid, and online environments while improving students’ learning outcomes, engagement, and motivation [21].

This study uses a comparative bibliometric analysis to investigate the role of AI in improving digital wellness among older people. Bibliometric analysis enables the quantification and systematic mapping of the existing literature, providing a structured review of academic studies in a specific domain [15]. Through this method, our research highlights key topics, influential studies, and emerging insights that underscore the importance of leveraging AI-driven innovations to create inclusive digital ecosystems. The findings highlight the importance of addressing challenges and developing solutions that enable older individuals to effectively navigate and manage their lives in an increasingly complex environment, as reflected in the growing body of research within the field of AI-oriented world [2,22]. The study analyzes the following research questions: (1) How has AI’s impact on digital wellness evolved for the general versus older populations? (2) What are the global collaboration patterns in AI research on digital wellness? (3) Which institutions lead in AI research for digital wellness? (4) What are the key journals and publication trends in this field? (5) What are the emerging themes in AI research for digital wellness?

## Methods

### Search Strategy

This study conducts a comparative bibliometric analysis to examine the role of AI in enhancing digital wellness for both the general population and older people, using the methodology outlined by Aria and Cuccurullo [23]. For this study, we followed a 3-phase approach: data collection, data analysis, and data visualization and reporting.

In the data collection phase, we queried, selected, and exported data from the Web of Science (WoS) core databases, focusing on publications from 2016 to 2025. The selected time period covers all publications in the field of study as indexed by WoS. WoS was preferred over alternatives such as Google Scholar or Scopus due to its higher quality bibliometric data, which is characterized by a lower rate of duplicate records [24] and broader coverage of high-impact journals [25]. We executed 2 search strings: SRCH_STR_ALL (which referred to the general population, excluding the older people) and SRCH_STR_OLD (which focused exclusively on the older population). These search strings were used to query the titles, keywords, and abstracts of all documents in the WoS collection (Textbox 1).

Textbox 1. **Search strings.**

SRCH_STR_ALL: (“AI” OR “Artificial Intelligence” OR “Machine Intelligence” OR “Intelligent Automation” OR “Smart Technology” OR “Automated Intelligence” OR “Algorithmic Intelligence”) **AND** (“Digital Wellness” OR “Digital Health” OR “e-Wellness” OR “Digital Wellbeing” OR “Technology-Enabled Wellness” OR “Digital Literacy” OR “Online Wellbeing”) **NOT** (“Elderly” OR “Senior*” OR “Older adult” OR “Mature adult” OR “Advanced in age” OR “aging” OR “Long-lived” OR “Retiree” OR “Golden ager”)

SRCH_STR_OLD: (“AI” OR “Artificial Intelligence” OR “Machine Intelligence” OR “Intelligent Automation” OR “Smart Technology” OR “Automated Intelligence” OR “Algorithmic Intelligence”) **AND** (“Digital Wellness” OR “Digital Health” OR “e-Wellness” OR “Digital Wellbeing” OR “Technology-Enabled Wellness” OR “Digital Literacy” OR “Online Wellbeing”) **AND** (“Elderly” OR “Senior*” OR “Older adult” OR “Aged” OR “Mature adult” OR “Advanced in age” OR “aging” OR “Long-lived” OR “Retiree” OR “Golden ager”)

### Data Analysis

We used both VOSviewer (version 1.6.20; Leiden University) [26] and Bibliometrix (The Bibliometrix R-package Development Team, University of Naples Federico II) [24] software to visually represent and assess the relationships between institutions, countries, authors, and keywords related to research on the use of AI to improve individuals’ digital wellness. Furthermore, some of the visualizations provide details about clusters emerging from these relationships. These clusters were formed using the VOSviewer algorithm, which groups related authors, keywords based on their connections, and proximity within the network.

### Bibliometric Analysis

**Figure 1.**
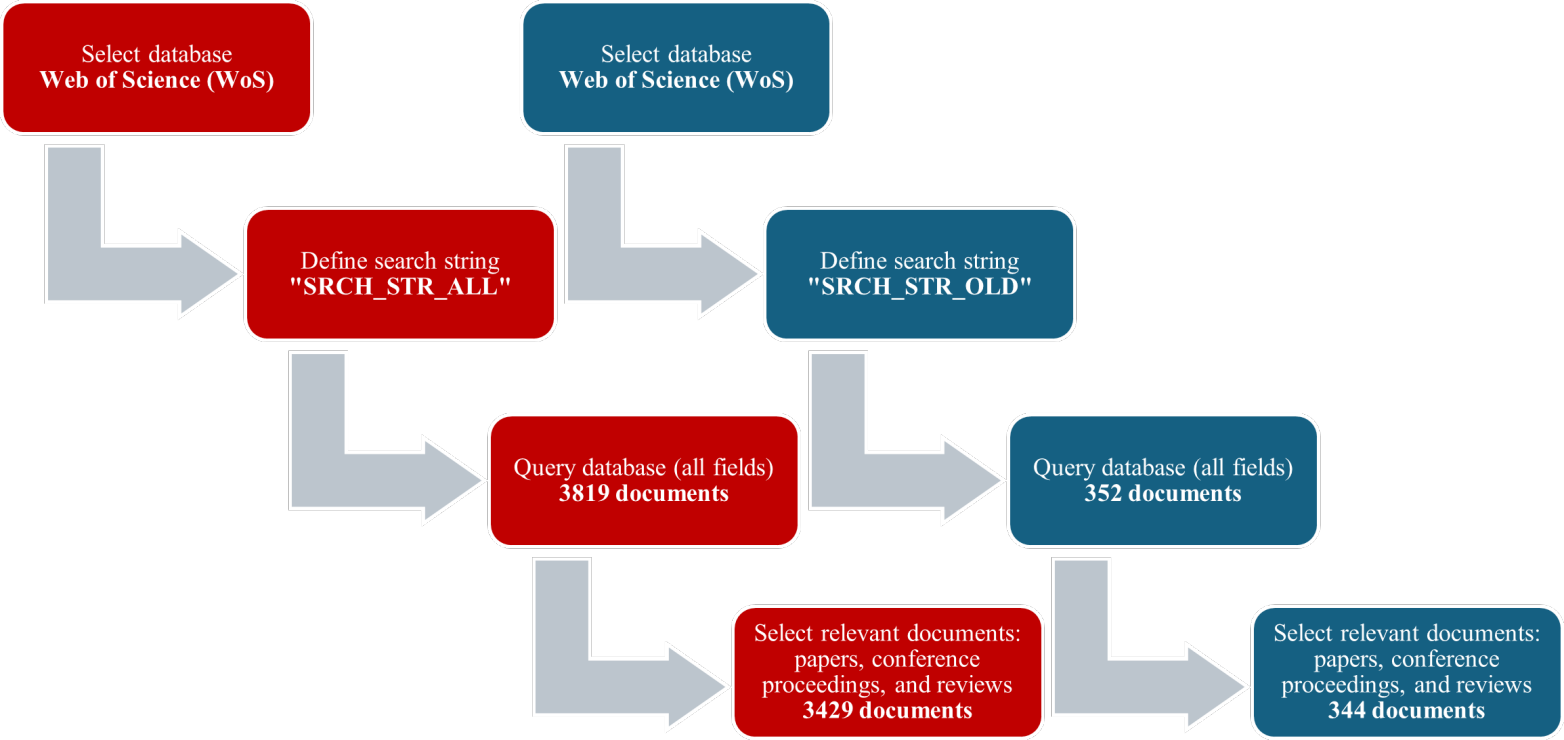
Summary of the data collection phase for both general and older populations.

The aforementioned search strings resulted in 3819 documents (352 related to older people), forming the initial datasets for this study. For quality assurance, only document types classified as papers, reviews, and proceeding papers were included, as these are most likely to have undergone a rigorous peer-review process before publication [27]. Consequently, editorial materials, letters, news items, meeting abstracts, and retracted publications were excluded from the dataset, yielding a final total of 3429 documents (344 related to older people) that were used for the bibliometric analysis. Figure 1 summarizes the data collection phase. The datasets comprise documents from 1171 sources (147 related to older people), authored by 18,911 individuals (2738 related to older people), and include a total of 47,044 unique references (4630 related to older people). The number of references per year was estimated by multiplying the average references per paper by the number of papers, with totals rounded for clarity.

### Ethical Considerations

This study did not require institutional review board or ethics committee approval, as it conducted a secondary bibliometric analysis of published literature from the WoS database without involving human participants, the collection of primary data, or access to identifiable information. Bibliometric and scientometric analyses that exclusively use publicly available, aggregated bibliographic metadata (publication records, author information, institutional affiliations, and citation indices) are generally exempt from ethics review requirements under standard institutional and international research ethics guidelines. This exemption is consistent with established policies of major research institutions and ethics oversight bodies. The Belmont Report and the Common Rule (45 CFR 46) in the United States define human subjects research as a systematic investigation designed to develop or contribute to generalizable knowledge involving human participants or identifiable private information. Since this research involved neither human participants nor access to identifiable individual data—only aggregate publication-level information already in the public domain—it falls outside the scope of research requiring ethics board oversight. Similarly, the Declaration of Helsinki and the International Council for Harmonisation guidelines recognize that research using nonidentifiable, aggregated data does not constitute human subjects research and therefore does not require ethics committee assessment. The European Union’s General Data Protection Regulation and comparable data protection frameworks exempt analyses of aggregated, anonymized bibliographic data from ethics review requirements, as such data cannot be traced to individual persons.

## Results

### Annual Publications, Citations, and Growth Forecast

#### Older Population

**Figure 2.**
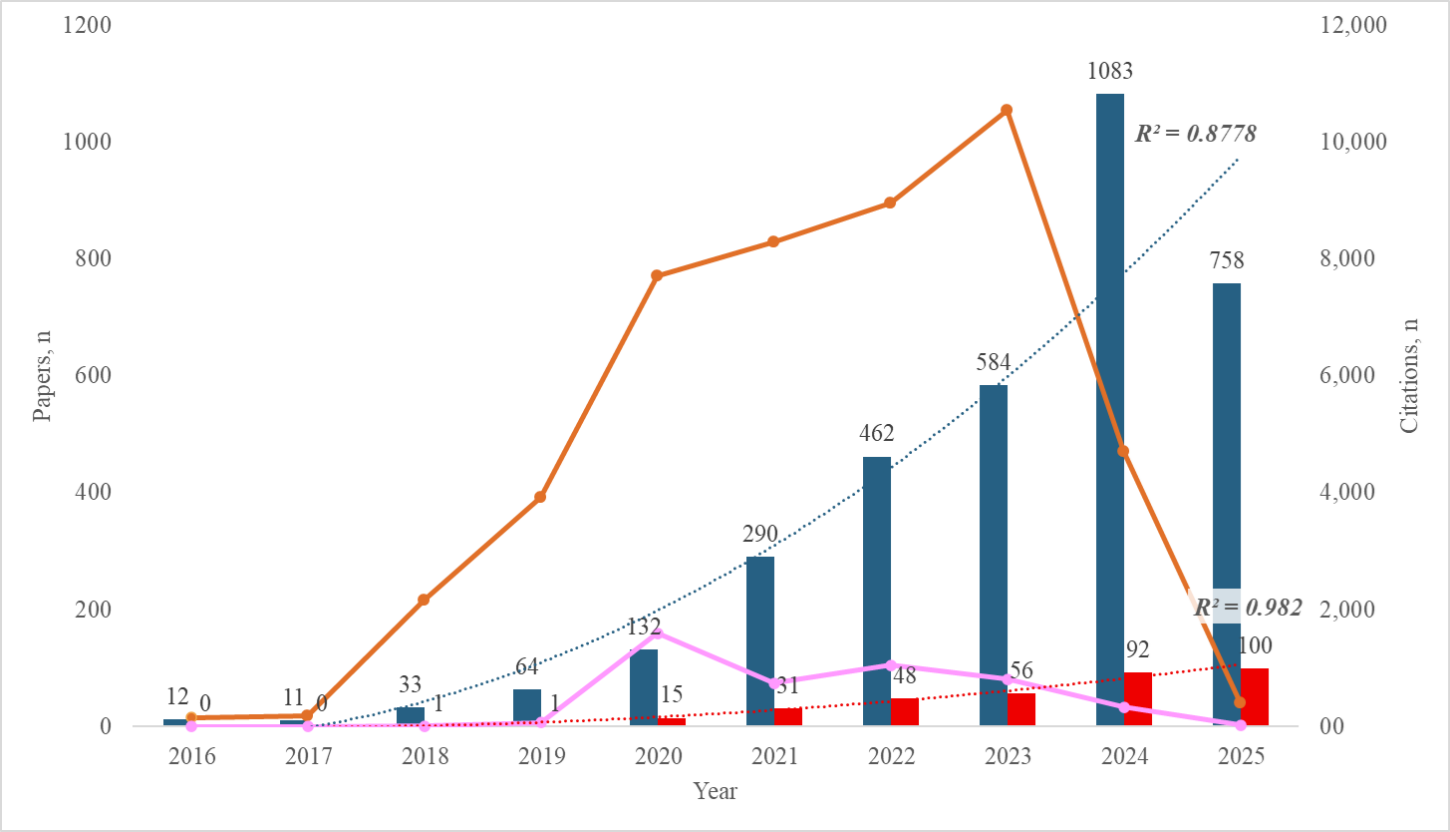
Publications and citations of artificial intelligence–focused research in improving digital wellness among both the general and older populations.

For the older population, the average annual number of papers published did not exceed 10 before 2022. After 2022, the output of papers increased and reached 100 in 2025. The results of the polynomial curve resembled those found among the general population and showed a very high coefficient of determination (*R*^2^=0.982). The average number of citations per paper was 13.5. Over the years, total citations per year fluctuated, reaching a peak at 1600 citations in 2020 and then decreased in the following years (Figure 2).

#### General Population

The average annual number of papers published for the general population did not exceed 100 before 2022. After 2022, the output of papers increased exponentially and reached 1083 in 2024. The polynomial curve was coherent with the yearly growth trend of literature, indicating a very high coefficient of determination (*R*^2^=0.8778). The average number of citations per paper was 13.7. Over the years, total citations per year fluctuated, reaching a peak at 10,547 citations in 2023 and then decreased to 4711 in 2024 and 409 in 2025.

The analysis revealed distinct publication trends when comparing the general and older populations. For the general population, the number of publications remained relatively low until 2022, after which there was a sharp increase, peaking in 2024. Despite this growth, citation patterns fluctuated, reaching a peak in 2023 before declining significantly in 2024 and 2025. In contrast, the older population had minimal research output prior to 2020. Following 2020, there was a notable increase in publications, reaching its highest point in 2025. Similarly, citations for papers on the older population peaked in 2024 before declining. Although both populations demonstrated exponential publication growth in recent years, the volume of research focused on older people remains smaller. However, citation trends for this group suggest a steadier and more consistent pattern compared to the more volatile citation trends observed in the general population.

### Countries

#### Older Population

**Figure 3.**
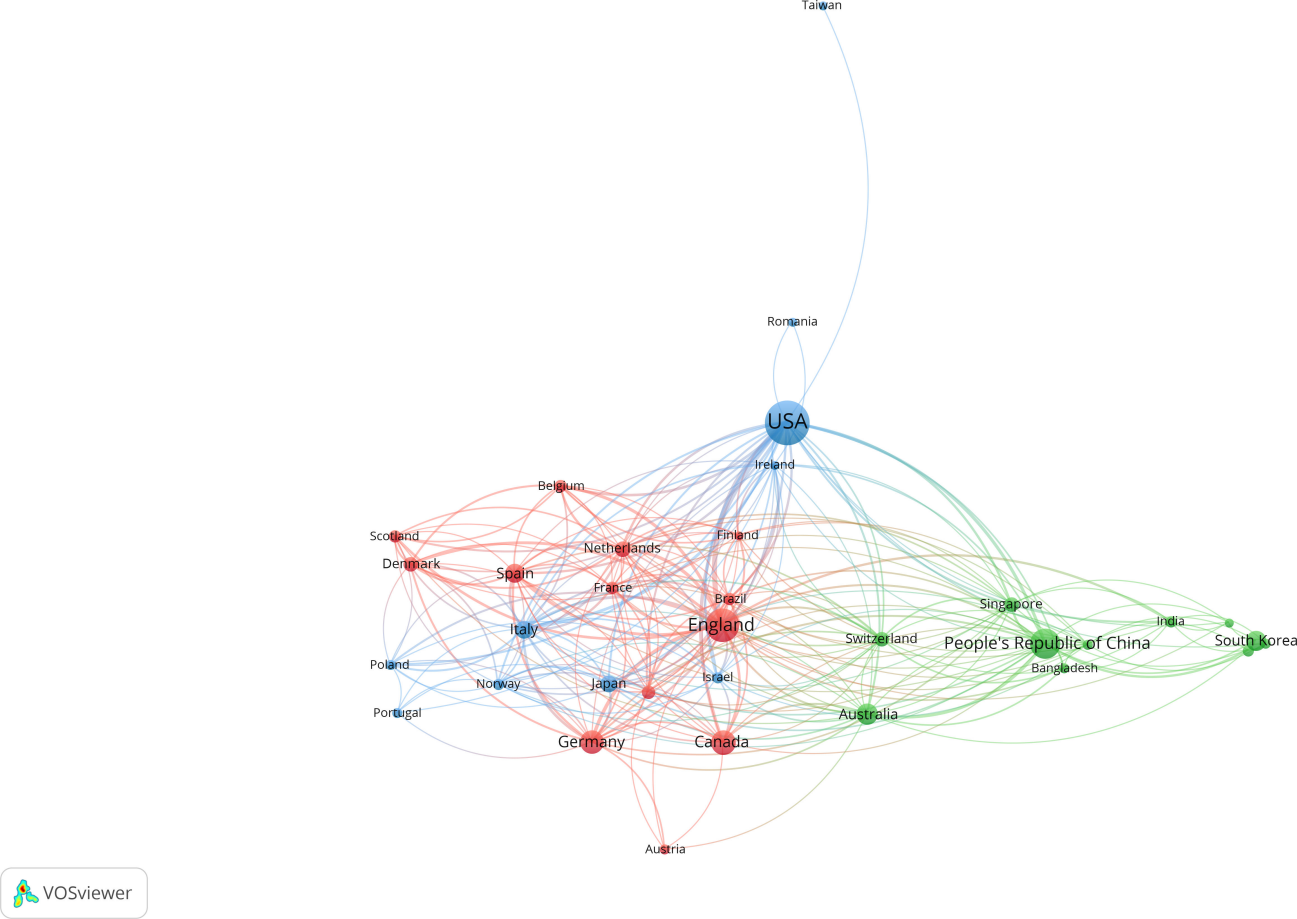
Network map of cooperation between countries for the older population. The size of dots represents a major hub of collaboration, and the different colors represent different clusters.

The majority of research papers on the topic of AI applications to improve well-being among older population came from the United States (79/344, 22.1%), China (40/344, 11.6%), the United Kingdom (38/344, 11.0%), Canada (22/344, 6.4%), Germany (20/344, 5.8%), and Spain (20/344, 5.8%). A network map of these countries was generated along with their allocation to clusters; in total, 34 countries published 5 or more papers on the aforementioned field (Figure 3). Cluster sizes were set to a minimum of 6 countries.

The network map revealed 3 clusters that highlighted international coauthorship in older people care research, with major hubs including the United States, China, and the United Kingdom leading collaborations. The United States was central with many global connections, while Germany, the United Kingdom, and China also created strong and steady regional clusters in Europe and Asia, respectively. European countries have close and internal relationships, and Canada, Brazil, Finland, and Israel are linked between the clusters, facilitating cross-regional partnerships. This structure shows a blend of regional collaborations and global partnerships centered around key research leaders.

#### General Population

**Figure 4.**
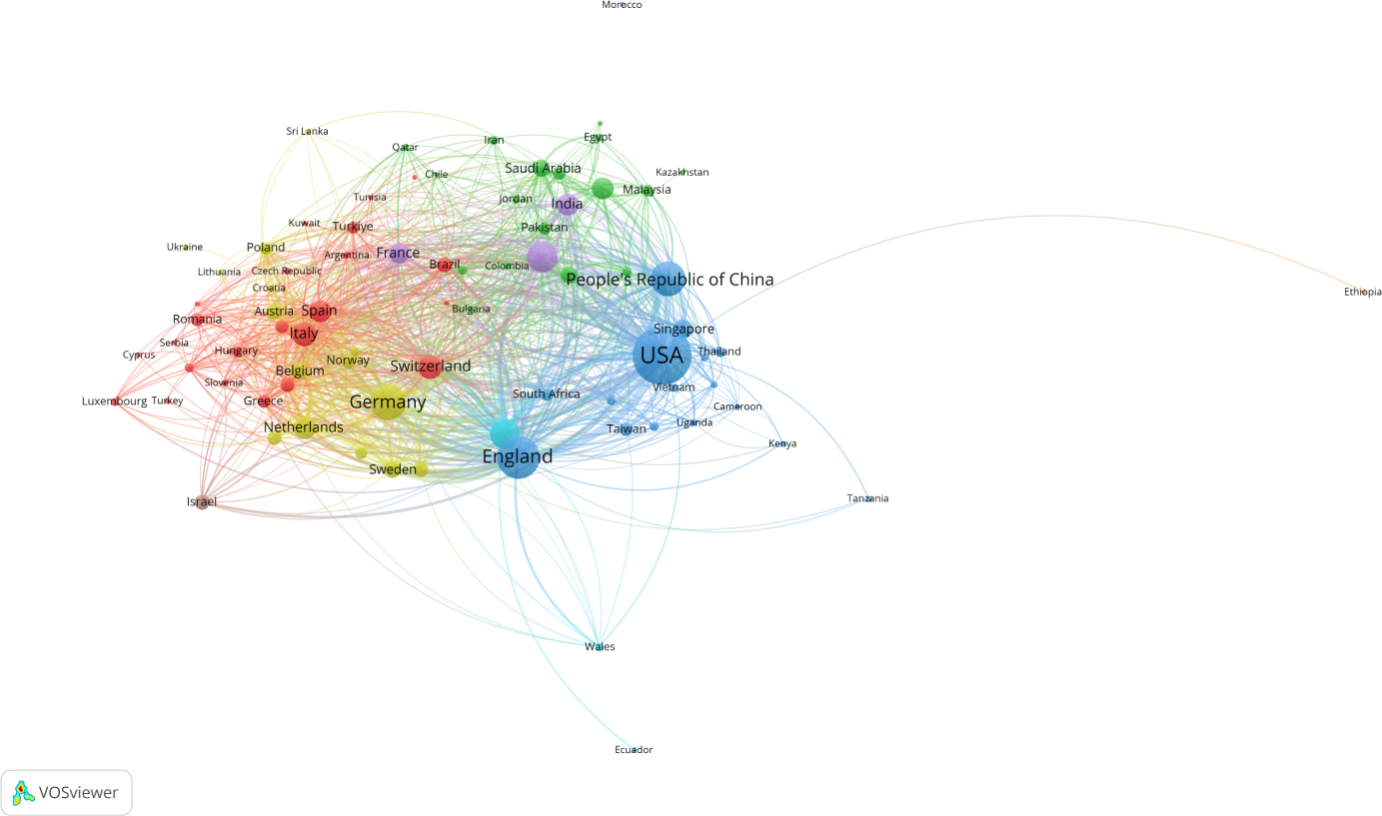
Network map of cooperation between countries for the general population. The size of dots represents a major hub of collaboration, and the different colors represent different clusters.

To form the network map between countries, we have calculated the number of publications based on the corresponding author’s country. Among the United States (671/3429, 19.5%), China (308/3429, 9.0%), the United Kingdom (304/3429, 8.9%), Germany (252/3429, 7.3%), Canada (175/3429, 5.1%), and Australia (167/3429, 4.9%), a network map between countries was generated. Based on this network map, several clusters of countries were revealed. In total, 82 countries met the threshold of publishing at least 5 papers (Figure 4). Cluster sizes were set to a minimum of 15 countries.

The coauthorship network map illustrates patterns of international cooperation, revealing four major clusters: (1) the United States, China, and the United Kingdom, along with several African and Asian countries (blue cluster); (2) Germany and other European and Asian or Middle Eastern countries (yellow cluster); (3) Canada with strong ties to Middle Eastern countries (green cluster); and (4) Italy, Spain, and a group of European countries (red cluster). The United States, China, the United Kingdom, Germany, Canada, Italy, and Spain lead in their respective clusters, reflecting their central roles in global research collaboration. In addition to these dominant hubs, several bridging countries play a key role in linking otherwise separate regions. Australia emerges as a central intermediary, connecting the Asian cluster (led by China) with Western nations such as the United States and the United Kingdom. South Africa also acts as a bridge, facilitating collaboration between the European cluster and other regions. Similarly, India connects both Western and Eastern networks, despite being rooted in the Asia-Pacific cluster. These bridging countries enhance global knowledge exchange and international integration, highlighting their significance not only in research output but also in fostering multiregional partnerships within the field. The research landscape for the general population was led by the United States, China, the United Kingdom, Germany, Canada, Italy, and Spain, forming 4 major clusters. These included broad collaborations between the United States, China, and the United Kingdom; a European Asian or Middle Eastern group centered around Germany; a Canadian-Middle Eastern cluster; and a European cluster led by Italy and Spain. In contrast, research on the older population revealed a more streamlined network structure, dominated by the United States, China, the United Kingdom, Germany, and Australia, and organized into 3 primary clusters. While both populations showed strong patterns of international collaboration, the general population network appeared more globally distributed, with several countries acting as bridges across clusters. Australia, South Africa, and India served as key intermediaries in the general network, enhancing connectivity between regions. In older people–focused research, bridging countries such as Canada, Brazil, Finland, and Israel played a similar integrative role, linking otherwise separate regional efforts. Although the United States remained a central hub in both domains, the general population network exhibited broader global integration, whereas older people research remained more concentrated within Europe and Asia.

### Coauthorship and Cocited Authors

While coauthorship means that authors form a cooperation between them, cocited authors mean that authors are cited together, not necessarily formed a direct cooperation between them. Following this nuance, we created both coauthorship and cocited authors maps, which provide information about potential collaborators and influential researcher groups.

#### Older Population

**Figure 5.**
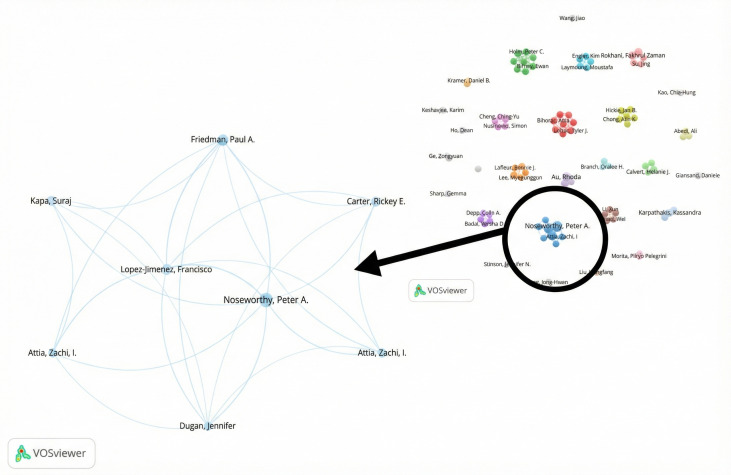
Network map of cooperation between authors for the older population. Each dot represents a different author, and each color represents a different allocation to a cluster.

A total of 2738 researchers participated in the research on the topic of AI applications for improving older population’s well-being, with 20 of 2738 (0.7%) publishing 3 or more studies on the topic. Peter A Noseworthy (5/344, 1.5%), and Ching-Yu Cheng, Paul A Friedman, Francisco Lopez-Himenez, Charumathi Sabanayagam, Yih-Chung Tham, and Tien Yin Wong with 4 of 344 (1.2%) publications each, published most papers. The coauthorship network visualization presented at the top of Figure 5 (including only authors with 2 or more publications) shows that while 95 authors met the publication threshold, only 8 were connected within a collaborative network, indicating a limited presence of high-yield, cooperative researchers in the field. Moreover, none of these prolific authors collaborated beyond their immediate groups, underscoring a scarcity of high-output researchers who actively engage in broader cooperative efforts. The structure is characterized by multiple disconnected clusters and minimal cross-group interaction, reflecting a fragmented and siloed research environment. The largest clusters—green, red, and blue—exhibit strong internal cohesion, while smaller clusters and dyads likely represent emerging collaborations or niche research communities. Although a few central authors within these clusters serve as bridges between otherwise isolated groups, their role is limited and does not compensate for the overall lack of widespread cooperation among top contributors. The color-coded clusters also imply thematic or institutional alignments. Structural holes between groups highlight potential opportunities for broader collaboration and knowledge integration. Overall, the network reflects a typical academic pattern—dominated by tightly knit research teams and limited peripheral engagement—resulting in a constrained diffusion of knowledge and slower overall development of the field.

**Figure 6.**
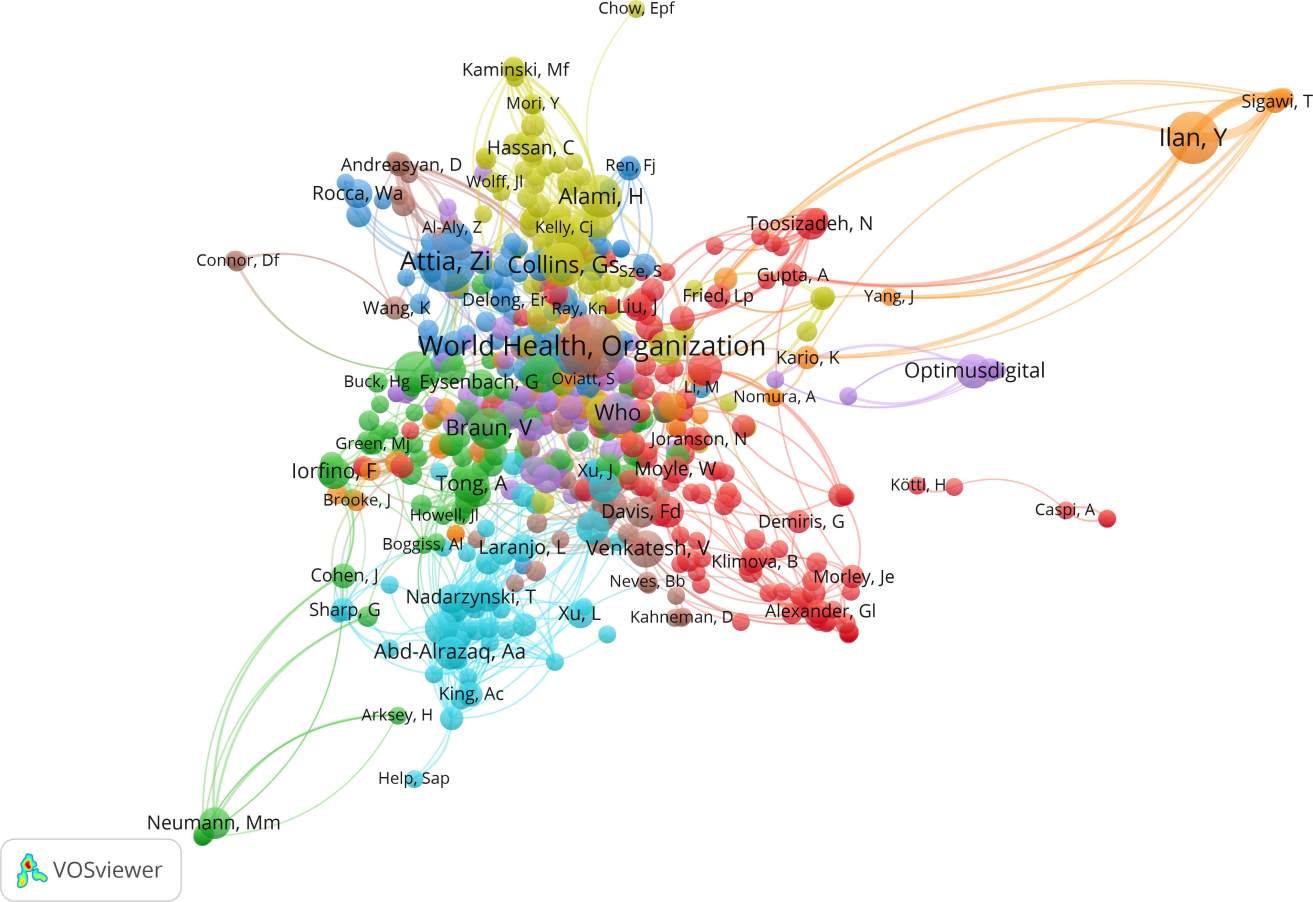
Network map of cocited authors for the older population. Each dot represents a different author, and each color represents a different allocation to a cluster. The arcs between clusters represent authors’ cocitations.

In addition, the author cocitation network (Figure 6) highlights the most influential research groups in the field. The top 5 cocited authors—World Health Organization, Yaron Ilan, Xiaoxuan Liu, Alaa Abd-Alrazaq, and Andrea Tricco—exhibited the strongest cocitation links, not only within their own clusters but also across other clusters. This pattern indicates their central role in shaping the intellectual structure of the research domain and fostering interdisciplinary influence.

#### General Population

A total of 18,911 researchers participated in the research on the aforementioned topic within the general population, with 937 (4.9%) of the authors publishing 3 or more studies; Leo Anthony Celi (23/3429, 0.7%), Paul A Friedman (19/3429, 0.6%), Peter A Noseworthy (17/3429, 0.5%), Zachi I Attia (15/3429, 0.4%), and Francisco Lopez-Jimenez, Bjoern M Eskofier, and Yaron Ilan with 14 of 3429 (0.4%) publications each published most of the papers.

**Figure 7.**
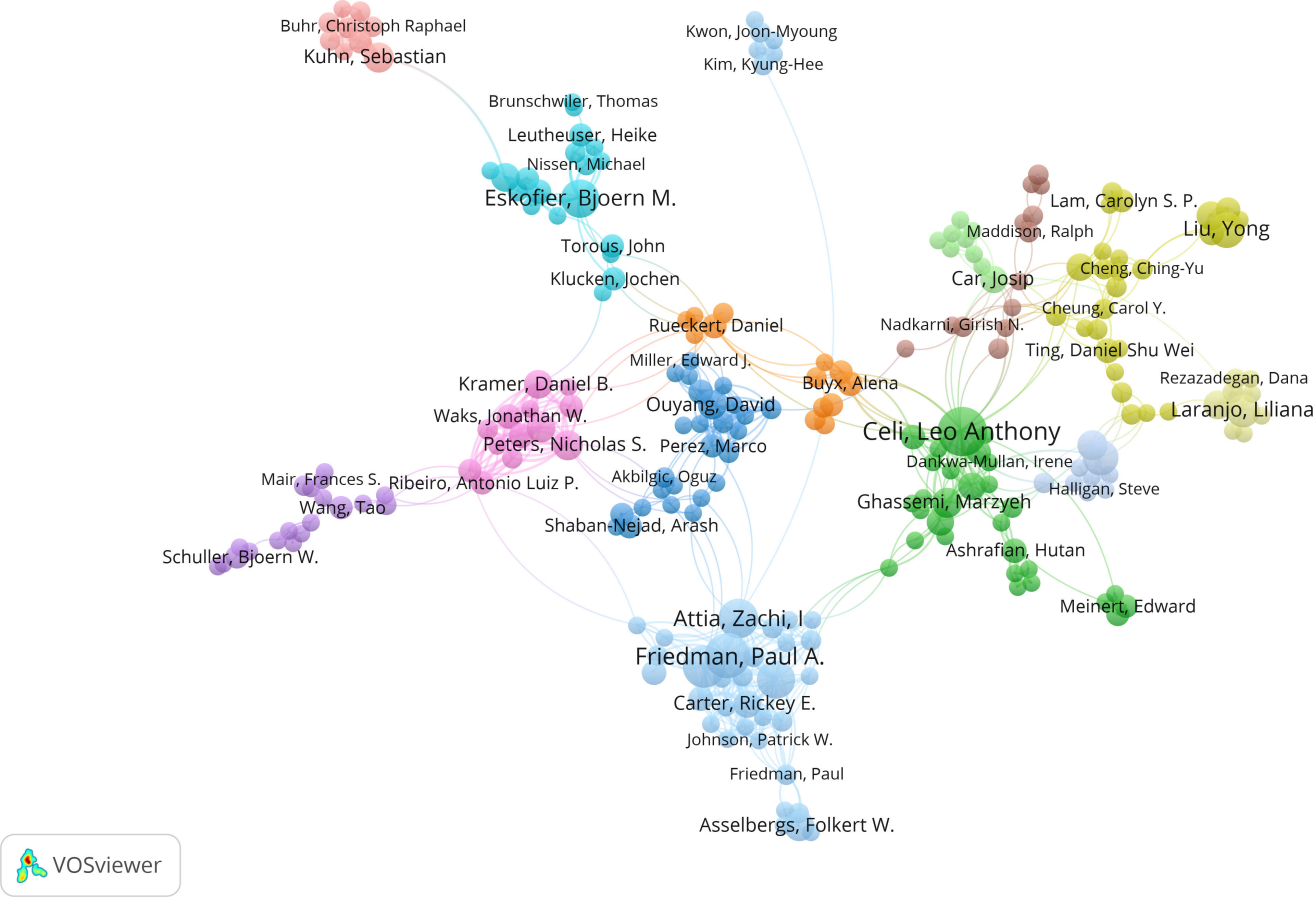
Network map of cooperation between authors for the general population. Each dot represents a different author, and each color represents a different allocation to a cluster.

The coauthorship network visualization in Figure 7, based on authors with 3 or more publications, reveals a moderately fragmented yet maturing research landscape. While 581 authors met the publication threshold, only 232 were connected within a collaborative network, indicating that many researchers remain siloed. The most prominent cluster centers around Paul A Friedman and Zachi I Attia, reflecting a well-established and productive research hub. Additional clusters led by Leo Anthony Celi (the United States), Yong Liu (Asia), and Bjoern M Eskofier (Europe) demonstrate strong regional and institutional collaborations. However, cross-cluster and international collaboration remains limited, with few authors acting as bridges between groups. The presence of several isolated nodes suggests emerging research directions or new contributors. Overall, the network reflects a field characterized by concentrated leadership and solid intragroup cooperation, yet with untapped potential for broader interdisciplinary and global integration.

**Figure 8.**
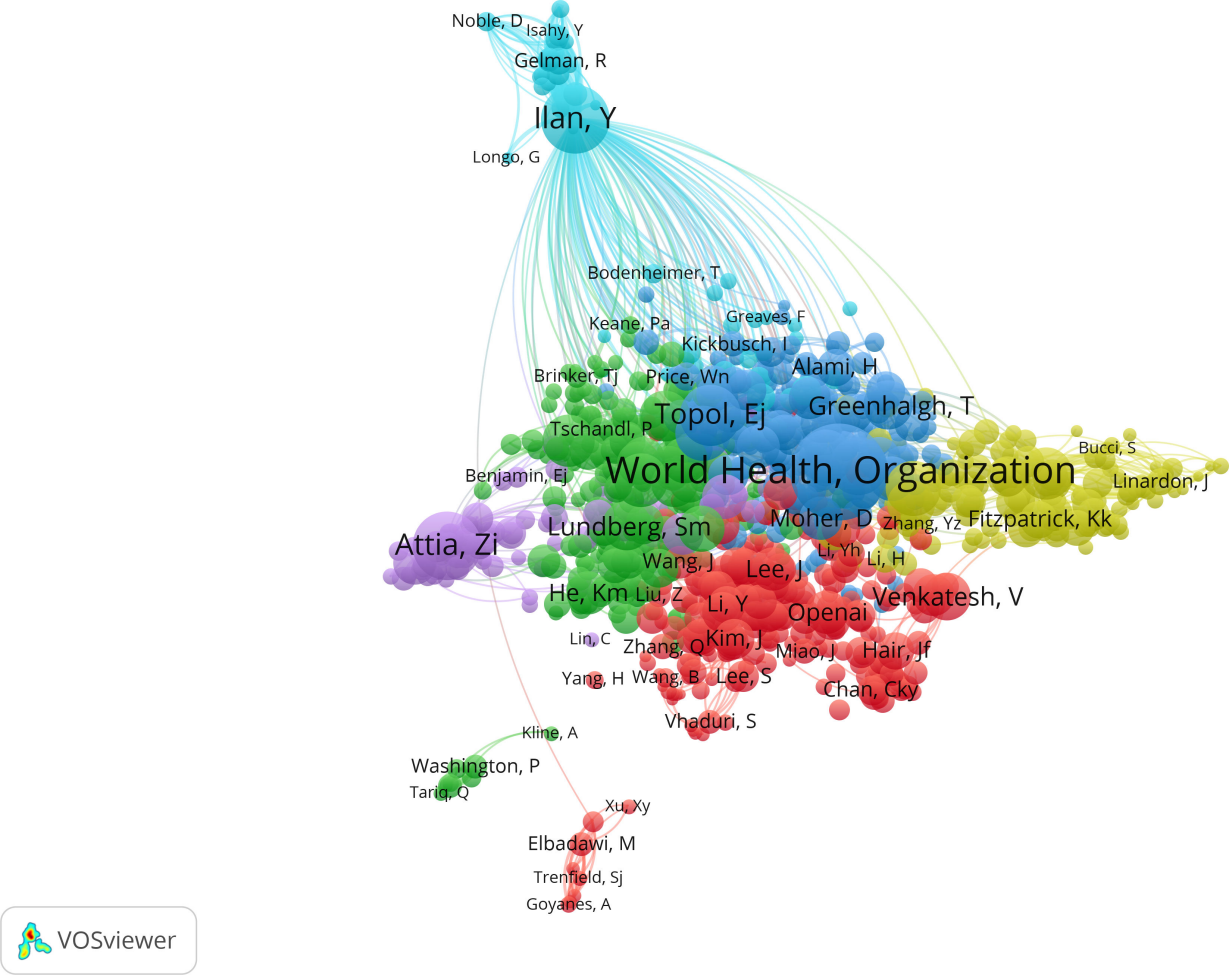
Network map of cocited authors for the general population. Each dot represents a different author, and each color represents a different allocation to a cluster. The arcs between clusters represent authors’ cocitations.

An analysis of the author cocitation network (Figure 8), with cluster sizes set to a minimum of 50 authors, reveals that several influential authors were cocited both within and across clusters, reflecting shared intellectual foundations and interdisciplinary relevance. Notably, Yaron Ilan, the World Health Organization, Eric J Topol, Zachi I Attia, and John Torous ranked among the top 5 most strongly cocited authors, serving as key intellectual bridges that connect distinct research communities.

### Institutions

#### Older Population

**Figure 9.**
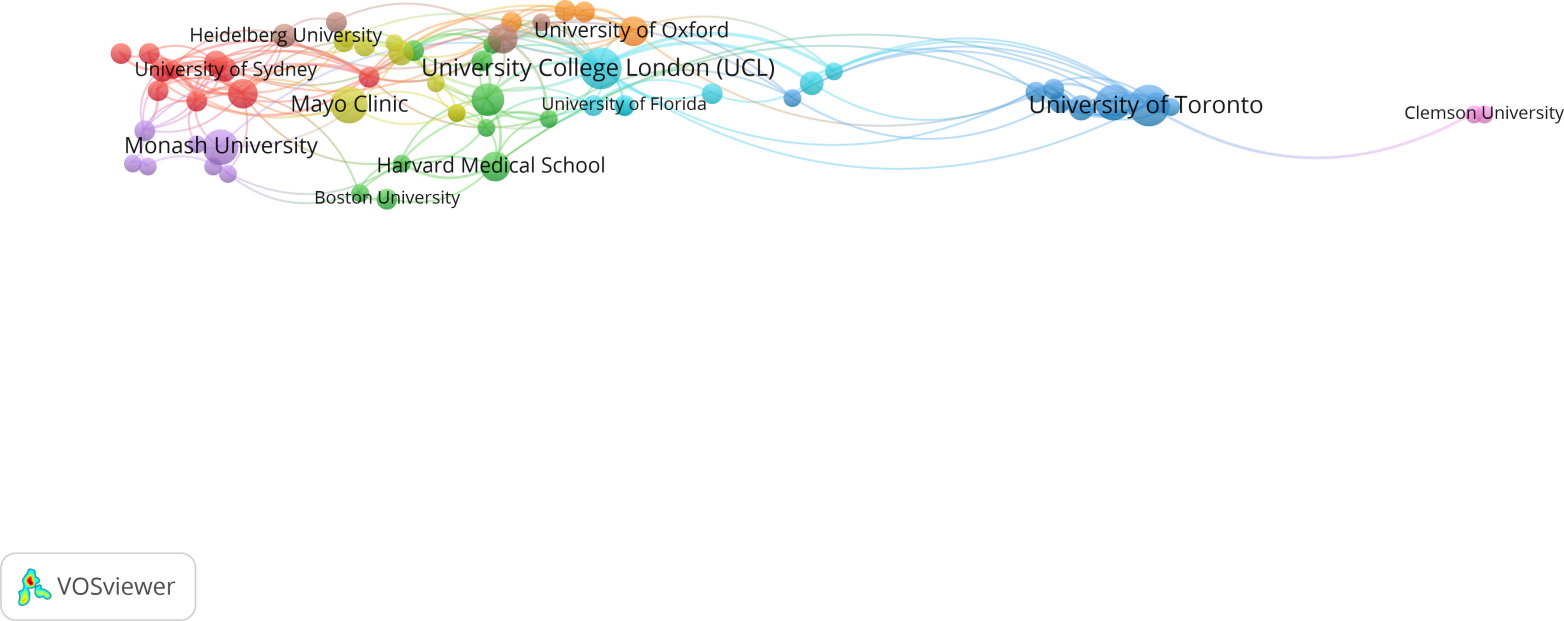
Network map of institutional collaboration in research on the older population. Each node represents a different institution, with colors indicating cluster membership. Arcs between nodes reflect coauthorship links, illustrating collaborative relationships within and across clusters.

A total of 757 institutions participated in relevant research on the role of AI in improving digital well-being among older population. The top 5 institutions involved in the research field were University of Toronto (77/757, 10.2%), University of London (69/757, 9.1%), National University of Singapore (48/757, 6.3%), University College London (48/757, 6.3%), and Harvard University (43/757, 5.7%). The network map of the institutions was generated and included 70 research institutions that published 3 or more papers; 62 institutions cooperated with other institutions (Figure 9).

#### General Population

**Figure 10.**
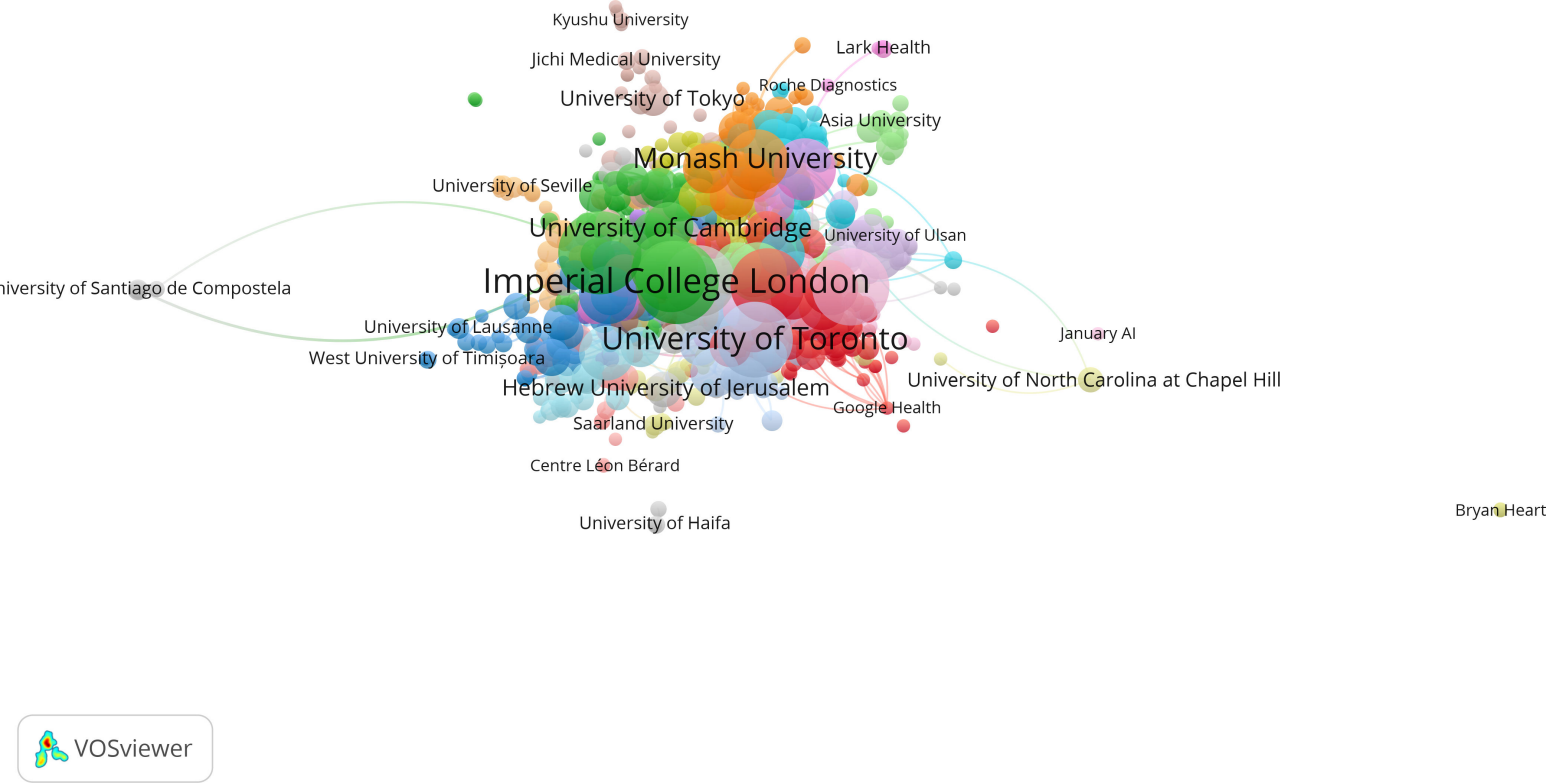
Network map of institutional collaboration in research on the general population. Each node represents a different institution, with colors indicating cluster membership. Arcs between nodes reflect coauthorship links, illustrating collaborative relationships within and across clusters.

A total of 3143 institutions participated in research on the role of AI in enhancing digital well-being. The top 5 institutions contributing to the field were the University of London (365/3143, 11.6%), Harvard University (316/3143, 10.1%), University of Toronto (281/3143, 8.9%), and both Mayo Clinic and University College London, with 219/3143 (7.0%) publications each. A network map of the institutions was generated, highlighting 900 research institutions that published 3 or more papers. Figure 10 illustrates 880 institutions actively collaborating with others.

### Journals

#### Older Population

In total, 344 papers were published in 147 journals. The top 5 journals that published the highest number of publications included *The Lancet Digital Health* (33/344, 9.6%), *Digital Health* (29/344, 8.4%), *The Journal of Medical Internet Research* (24/344, 7.0%), and *European Heart Journal—Digital Health*, *The Journal of Medical Internet Research Aging*, and *The Journal of Medical Internet Research Formative Research*, with 14/344 (4.1%) publications each.

Next, we have analyzed both research areas and categories. While research areas are broader, high-level groupings that reflect general fields of studies, categories are more specific and detailed classifications that group journals and publications into specialized fields.

To analyze the publication’s research areas and categories, only research areas and categories with at least 5 and 10 publications, respectively, were included. Results revealed that of the 344 papers, the leading fields (Table 1) were medical informatics (163/344, 47.4%), health care sciences services (129/344, 37.5%), public environmental occupational health (58/344, 16.9%), general internal medicine (44/344, 12.8%), and geriatrics and gerontology (24/344, 7.0%). As for the research categories, the top 5 categories as defined by the WoS are medical informatics (163/344, 47.4%), health care science services (127/344, 36.9%), public environmental occupational health (58/344, 16.9%), medicine general internal (44/344, 12.8%), and health policy services (37/344, 10.8%; Table 2).

**Table 1.** Classification of research paper areas or categories for the older population.

Research areas	Papers (n=344), n (%)
Medical informatics	163 (47.4)
Health care science services	129 (37.5)
Public environmental occupational health	58 (16.9)
General internal medicine	44 (12.8)
Geriatrics, gerontology	32 (9.3)
Computer science	31 (9.0)
Engineering	24 (7.0)
Cardiovascular system, cardiology	23 (6.7)
Neurosciences, neurology	11 (3.2)
Science technology, other topics	10 (2.9)
Education, educational research	7 (2.0)
Environmental sciences, ecology	5 (1.5)
Oncology	4 (1.2)
Telecommunications	4 (1.2)
Chemistry	3 (0.9)
Nursing	3 (0.9)
Pharmacology, pharmacy	3 (0.9)
Physics	3 (0.9)
Psychiatry	3 (0.9)
Psychology	3 (0.9)
Rehabilitation	3 (0.9)
Research experimental medicine	3 (0.9)
Biotechnology, applied microbiology	2 (0.6)
Endocrinology metabolism	2 (0.6)
Information science, library science	2 (0.6)
Linguistics	2 (0.6)
Materials science	2 (0.6)
Radiology, nuclear medicine, medical imaging	2 (0.6)
Social sciences, other topics	2 (0.6)
Surgery	2 (0.6)
Automation control systems	1 (0.3)
Behavioral sciences	1 (0.3)
Biochemistry, molecular biology	1 (0.3)
Business, economics	1 (0.3)
Cell biology	1 (0.3)
Construction building technology	1 (0.3)
Cultural studies	1 (0.3)
Gastroenterology, hepatology	1 (0.3)
Instruments, instrumentation	1 (0.3)
Life sciences, biomedicine, other topics	1 (0.3)
Literature	1 (0.3)
Mathematical computational biology	1 (0.3)
Obstetrics, gynecology	1 (0.3)
Ophthalmology	1 (0.3)
Orthopedics	1 (0.3)
Pediatrics	1 (0.3)
Public administration	1 (0.3)
Robotics	1 (0.3)
Sport sciences	1 (0.3)

**Table 2.** Classification of paper categories for the older population.

Web of Science categories	Papers (n=344), n (%)
Medical informatics	163 (47.4)
Health care science services	127 (36.9)
Public environmental occupational health	58 (16.9)
Medicine general internal	44 (12.8)
Health policy services	37 (10.8)
Geriatrics, gerontology	29 (8.4)
Gerontology	24 (7.0)
Cardiac, cardiovascular systems	20 (5.8)
Computer science, interdisciplinary applications	17 (4.9)
Computer science, theory methods	12 (3.5)
Engineering biomedical	12 (3.5)
Computer science, artificial intelligence	9 (2.6)
Neurosciences	9 (2.6)
Multidisciplinary sciences	8 (2.3)
Computer science, cybernetics	6 (1.7)
Computer science, information systems	6 (1.7)
Engineering electrical electronic	6 (1.7)
Education, educational research	5 (1.5)
Environmental sciences	5 (1.5)
Engineering multidisciplinary	4 (1.2)
Oncology	4 (1.2)
Telecommunications	4 (1.2)
Education, scientific disciplines	3 (0.9)
Medicine research experimental	3 (0.9)
Nursing	3 (0.9)
Peripheral vascular disease	3 (0.9)
Pharmacology, pharmacy	3 (0.9)
Physics applied	3 (0.9)
Psychiatry	3 (0.9)
Rehabilitation	3 (0.9)
Biotechnology, applied microbiology	2 (0.6)
Chemistry multidisciplinary	2 (0.6)
Clinical neurology	2 (0.6)
Computer science, hardware architecture	2 (0.6)
Endocrinology metabolism	2 (0.6)
Environmental studies	2 (0.6)
Ergonomics	2 (0.6)
Green sustainable science technology	2 (0.6)
Information science, library science	2 (0.6)
Linguistics	2 (0.6)
Materials science multidisciplinary	2 (0.6)
Psychology multidisciplinary	2 (0.6)
Radiology, nuclear medicine, medical imaging	2 (0.6)
Social sciences interdisciplinary	2 (0.6)
Surgery	2 (0.6)
Automation control systems	1 (0.3)
Behavioral sciences	1 (0.3)
Biochemistry, molecular biology	1 (0.3)
Biology	1 (0.3)
Cell biology	1 (0.3)
Chemistry analytical	1 (0.3)
Construction building technology	1 (0.3)
Cultural studies	1 (0.3)
Economics	1 (0.3)
Engineering civil	1 (0.3)
Engineering industrial	1 (0.3)
Gastroenterology, hepatology	1 (0.3)
Instruments, instrumentation	1 (0.3)
Language linguistics	1 (0.3)
Literature romance	1 (0.3)
Mathematical computational biology	1 (0.3)
Obstetrics, gynecology	1 (0.3)
Ophthalmology	1 (0.3)
Orthopedics	1 (0.3)
Pediatrics	1 (0.3)
Psychology	1 (0.3)
Psychology developmental	1 (0.3)
Public administration	1 (0.3)
Robotics	1 (0.3)
Sport sciences	1 (0.3)

#### General Population

The 3429 included papers were published in 1171 journals. The top 5 journals that published the highest number of publications included *Digital Health* (331/3429, 9.7%), *Frontiers in Digital Health* (250/3429, 7.3%), *Journal of Medical Internet Research* (185/3429, 5.4%), *PLOS Digital Health* (151/3429, 4.4%), and *Lancet Digital Health* (143/3429, 4.2%).

When examining the broad research areas of these publications, only those with at least 45 related publications were considered. The leading fields (Table 3) include medical informatics (1411/3429, 41.4%), health care science services (1357/3429, 39.8%), computer science (544/3429, 15.9%), public environmental occupational health (506/3429, 14.8%), and general internal medicine (305/3429, 8.9%). Specifically, when analyzing publication source categories with at least 100 associated publications (Multimedia Appendix 1), the top 5 categories as defined by WoS are medical informatics (1411/3429, 41.4%), health care science services (1339/3429, 39.3%), public environmental occupational health (506/3429, 14.8%), health policy services (401/3429, 11.8%), and medicine general internal (298/3429, 8.7%).

**Table 3.** Classification of paper research areas for the general population.

Research areas	Papers (n=3429), n (%)
Medical informatics	1411 (41.4)
Health care science services	1357 (39.8)
Computer science	544 (16.0)
Public environmental occupational health	506 (14.8)
General internal medicine	305 (8.9)
Engineering	281 (8.2)
Cardiovascular system, cardiology	233 (6.8)
Education, educational research	175 (5.1)
Science technology, other topics	88 (2.6)
Pharmacology, pharmacy	49 (1.4)
Chemistry	48 (1.4)
Psychiatry	47 (1.4)
Telecommunications	45 (1.3)
Neurosciences, neurology	42 (1.2)
Oncology	40 (1.2)
Psychology	39 (1.1)
Business, economics	37 (1.1)
Environmental sciences, ecology	37 (1.1)
Information science, library science	37 (1.1)
Social sciences, other topics	37 (1.1)
Surgery	35 (1.0)
Instruments, instrumentation	34 (1.0)
Endocrinology and metabolism	32 (0.9)
Nursing	31 (0.9)
Biotechnology, applied microbiology	29 (0.9)
Radiology, nuclear medicine, medical imaging	29 (0.9)
Research experimental medicine	28 (0.8)
Communication	26 (0.8)
Physics	24 (0.7)
Pediatrics	22 (0.7)
Genetics, heredity	18 (0.5)
Biochemistry, molecular biology	17 (0.5)
Materials science	17 (0.5)
Gastroenterology, hepatology	16 (0.5)
Mathematical computational biology	16 (0.5)
Infectious diseases	15 (0.4)
Linguistics	15 (0.4)
Rheumatology	15 (0.4)
Government law	12 (0.4)
Mathematics	12 (0.4)
Rehabilitation	11 (0.3)
Cell biology	10 (0.3)
Public administration	10 (0.3)
Robotics	10 (0.3)
Dentistry, oral surgery medicine	9 (0.3)
Medical ethics	9 (0.3)
Medical laboratory technology	9 (0.3)
Operations research, management science	9 (0.3)
Ophthalmology	9 (0.3)
Otorhinolaryngology	9 (0.3)
Urology, nephrology	9 (0.3)
Arts, humanities, other topics	8 (0.2)
Biomedical social sciences	8 (0.2)
Biophysics	8 (0.2)
Geriatrics, gerontology	8 (0.2)
Sociology	8 (0.2)
Immunology	7 (0.2)
Orthopedics	7 (0.2)
Sport sciences	7 (0.2)
Physiology	6 (0.2)
Social issues	6 (0.2)
Agriculture	5 (0.2)
Allergy	5 (0.2)
Automation control systems	5 (0.2)
Dermatology	5 (0.2)
Food science technology	5 (0.2)
Imaging science, photographic technology	5 (0.2)
Nutrition dietetics	5 (0.2)
Obstetrics, gynecology	5 (0.2)
Respiratory system	5 (0.2)
Tropical medicine	5 (0.2)
Life sciences, biomedicine, other topics	4 (0.1)
Parasitology	4 (0.1)
Acoustics	3 (0.1)
Energy, fuels	3 (0.1)
Hematology	3 (0.1)
Microbiology	3 (0.1)
Toxicology	3 (0.1)
Transplantation	3 (0.1)
Anesthesiology	2 (0.1)
Behavioral sciences	2 (0.1)
Construction building technology	2 (0.1)
Cultural studies	2 (0.1)
Development studies	2 (0.1)
Electrochemistry	2 (0.1)
History, philosophy of science	2 (0.1)
International relations	2 (0.1)
Legal medicine	2 (0.1)
Optics	2 (0.1)
Philosophy	2 (0.1)
Remote sensing	2 (0.1)
Social work	2 (0.1)
Anthropology	1 (0.0)
Developmental biology	1 (0.0)
Emergency medicine	1 (0.0)
Ethnic studies	1 (0.0)
Forestry	1 (0.0)
History	1 (0.0)
Mechanics	1 (0.0)
Metallurgy, metallurgical engineering	1 (0.0)
Meteorology, atmospheric sciences	1 (0.0)
Mining, mineral processing	1 (0.0)
Pathology	1 (0.0)
Substance abuse	1 (0.0)
Transportation	1 (0.0)
Urban studies	1 (0.0)
Virology	1 (0.0)
Water resources	1 (0.0)

When analyzing scientific publications for both the general and older populations, notable differences emerged in research scope and publication volume. Research on the general population included 3429 papers across 1171 journals, with top outlets such as *Digital Health*, *Frontiers in Digital Health*, and *Journal of Medical Internet Research* accounting for a substantial share. The leading research areas were medical informatics, health care sciences service, and computer science, while the most prominent publication categories—according to WoS—were medical informatics, health care science services, and public environmental occupational health, alongside health policy services and general internal medicine.

In contrast, older people–focused literature comprised 344 papers published in 147 journals, with *The Lancet Digital Health* and *Digital Health* emerging as the most frequent sources. Despite the lower volume, research on older people emphasized similar domains, particularly medical informatics and health care science services. However, it placed relatively more emphasis on public environmental occupational health, geriatrics and gerontology, and general internal medicine. The category distribution further highlighted the relevance of health policy services for aging populations, suggesting a research shift toward addressing the specific health and policy needs of older adults.

### Co-Occurrence Keywords

#### Older Population

**Figure 11.**
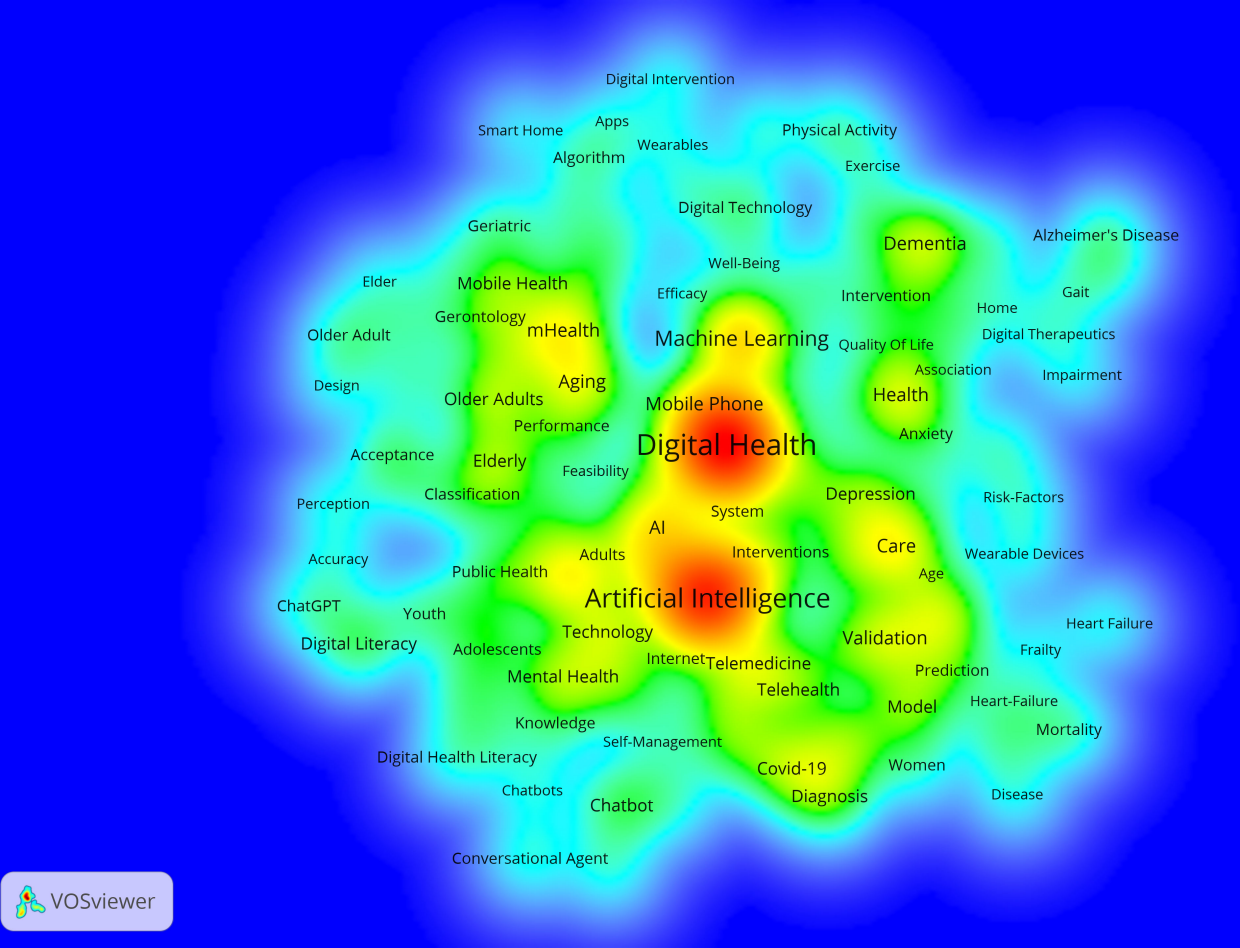
Density map of keywords related to the role of artificial intelligence in improving older population’s well-being. In the density map, warm colors (ie, red) represent more frequent keywords, and cold colors (ie, cyan) represent less frequent keywords.

The data included a total of 2102 keywords. Our analysis included both authors’ keywords and WoS’s Keyword Plus. The main high-frequency keywords included “artificial intelligence” (154/2102, 7.3%), “digital health” (126/2102, 6.0%), “machine learning” (37/2102, 1.8%), “mhealth” and “care with” 24 of 2102 (1.1%) times each, and “dementia” (22/2102, 1.1%). Since the topic of AI was represented by quite similar keywords (ie, “artificial intelligence,” “artificial-intelligence,” and “ai”), they were taken together when counting keywords’ frequency. Cluster analysis was carried out on 120 keywords with a frequency of 5 or more, and they were finally clustered into 4 groups (Figure 11). Cluster sizes were set to a minimum of 15 keywords.

**Figure 12.**
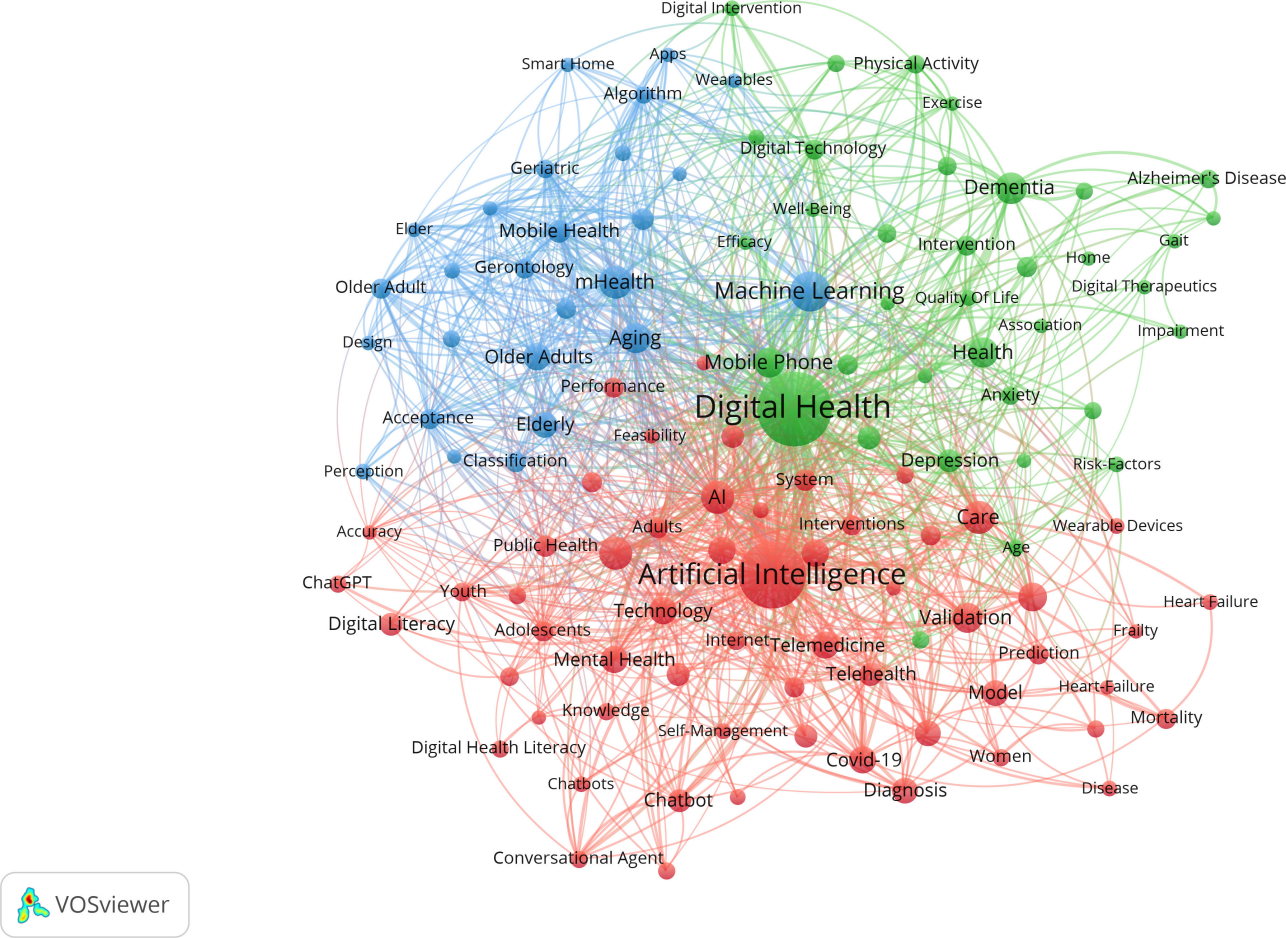
Network map of keywords related to the role of artificial intelligence in improving older populations’ well-being. In the network map, the size of dots represents their frequency, and each color represents a different allocation to a cluster.

When analyzing the clusters produced, results reveal a comprehensive view of the multifaceted nature of DH and its intersections with health care delivery and the needs of aging populations. Furthermore, the analysis shows a complex reciprocity between medication, technology, and human behavior factors. Among older populations, mobile health (mHealth) and dementia were emphasized, as well as risk management and chronic conditions, indicating challenges related to this specific population (Figure 12).

#### General Population

**Figure 13.**
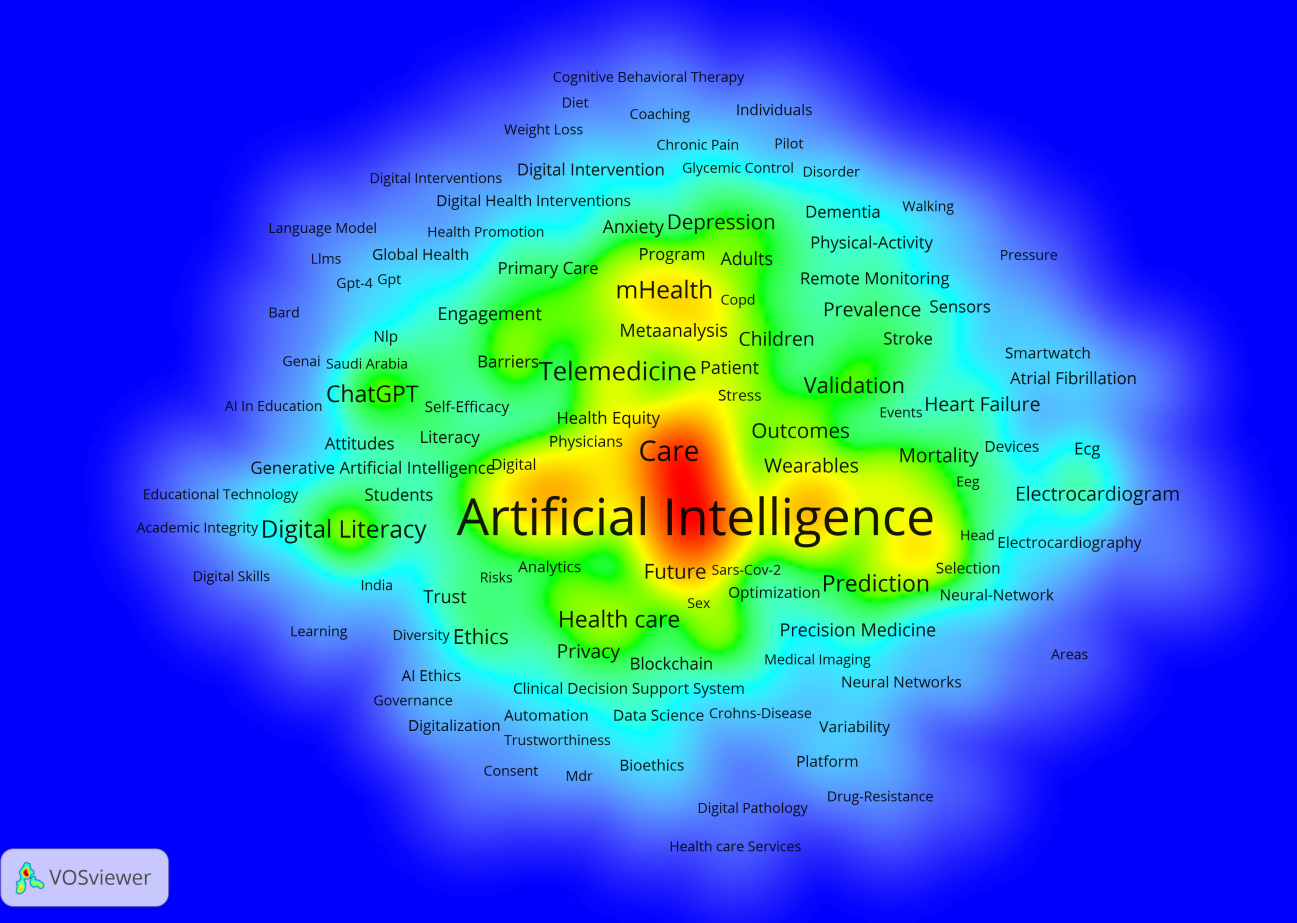
Density map of keywords related to the role of artificial intelligence in improving general population’s well-being. In the density map, warm colors (ie, red) represent more frequent keywords, and cold colors (ie, cyan) represent less frequent keywords.

The data included a total of 11,473 keywords. The main high-frequency keywords included “artificial intelligence” (1678/11,473, 14.6%), “digital health” (990/11,473, 8.6%), “machine learning” (436/11,473, 3.8%), “care” (224/11,473, 2.0%), “deep learning” (189/11,473, 1.7%), “health” (184/11,473, 1.6%), “technology” (164/11,473, 1.4%), “telemedicine” (156/11,473, 1.4%), and “digital literacy” (150/11,473, 1.3%). Similar to the older population, all AI keywords were taken together. Cluster analysis was carried out on 901 keywords with a frequency of 5 or more, and they were finally clustered into 6 groups (Figure 13). Cluster sizes were set to a minimum of 85 keywords.

**Figure 14.**
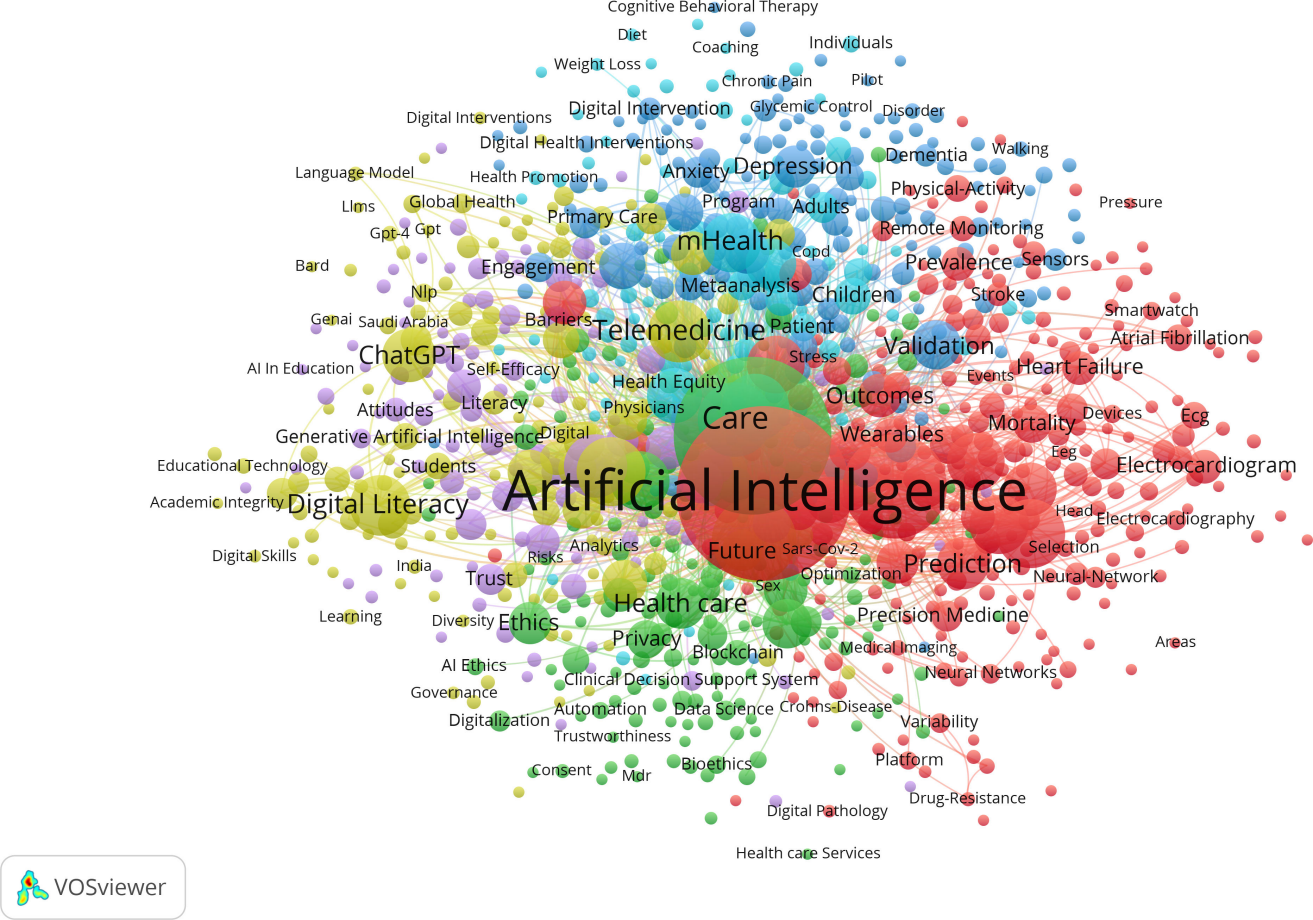
Network map of keywords related to the role of artificial intelligence in improving general population’s well-being. In the network map, the size of dots represents their frequency, and each color represents a different allocation to a cluster.

Further investigation of the created clusters reveals the broad and complex nature of how AI was being applied and integrated within the health care and DH landscapes, touching on medical, technological, ethical, and practical considerations. Key themes like “electronic health,” “tele medicine,” “depression,” and “cardiovascular diseases” further underscore the diverse applications and impacts of AI in these domains (Figure 14).

### Theme Evolution

#### Older Population

**Figure 15.**
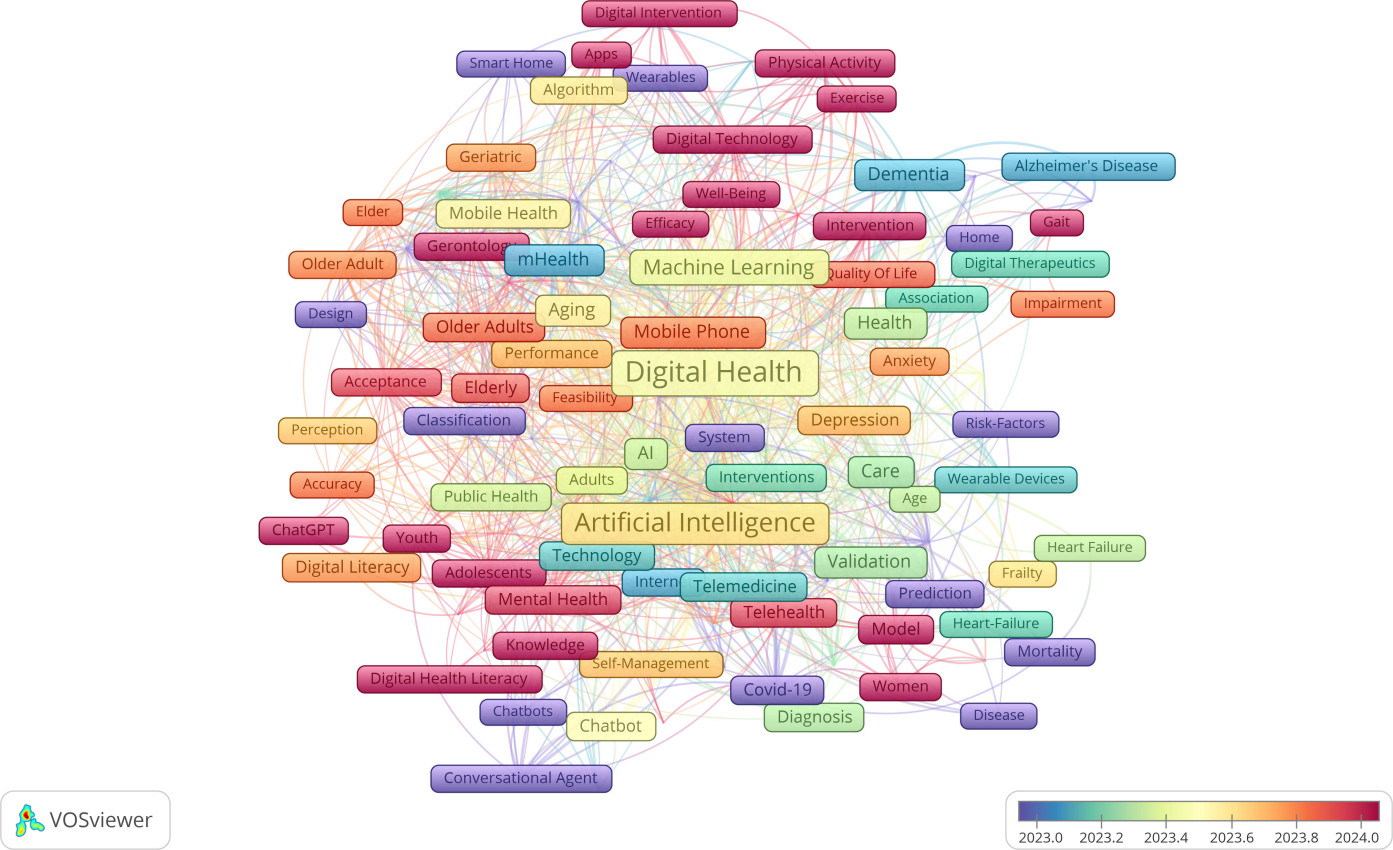
Time overlay network map of the role of artificial intelligence in improving older population’s well-being.

The visual overlay (Figure 15) map reveals a dynamic evolution of older people–related research. While earlier literature emphasized foundational themes such as “aging,” “frailty,” and “chronic illness,” recent years (2023-2024) have seen a noticeable shift toward digital and psychosocial aspects. Keywords like “telemedicine,” “social isolation,” and “digital health” emerged as central nodes with higher average publication years, indicating growing scholarly interest in technology-enabled solutions for aging populations. The clustering further demonstrates the interdisciplinary nature of this domain, spanning public health, social sciences, and digital innovation.

**Figure 16.**
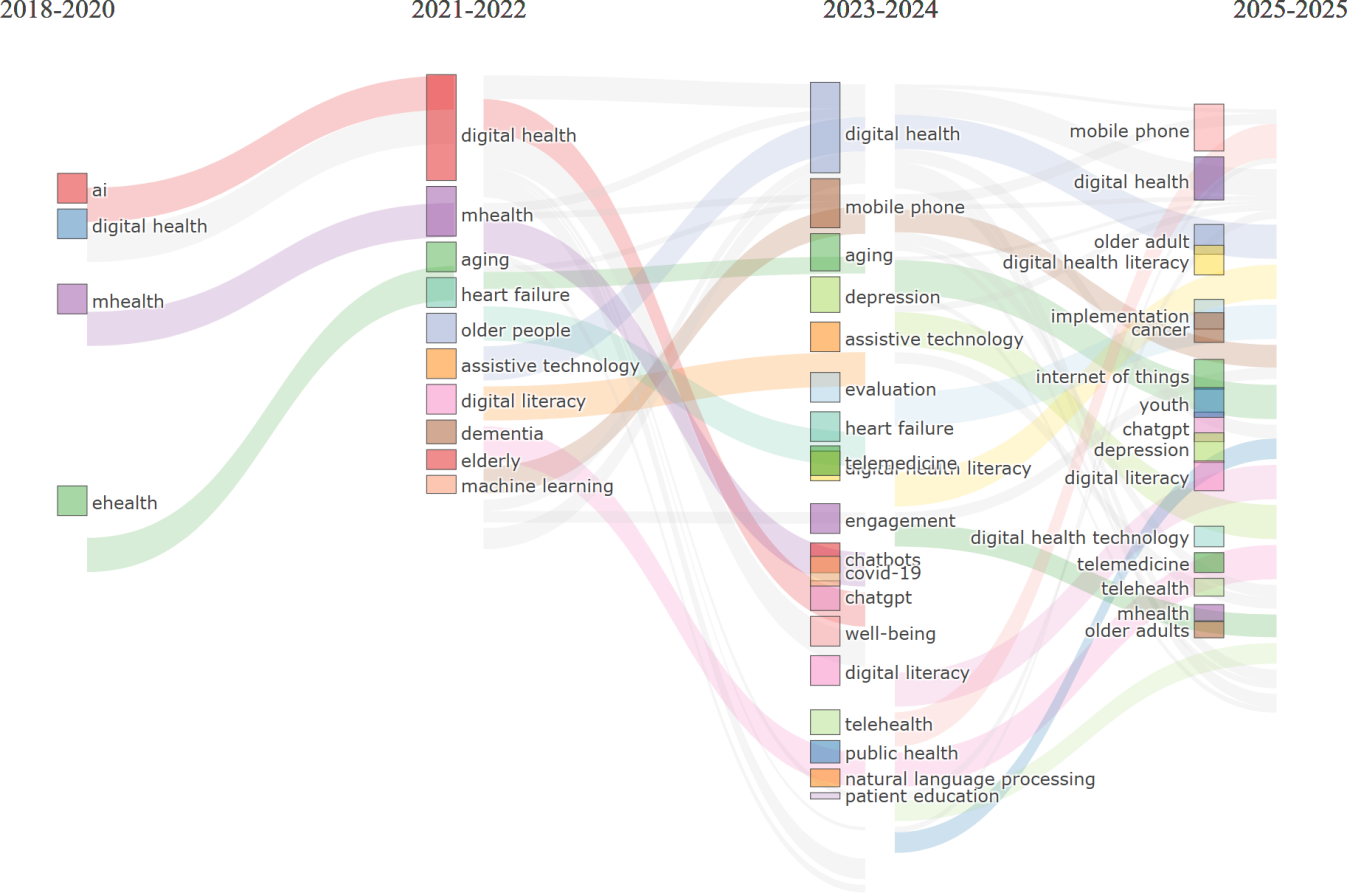
The thematic evolution map for the role of AI in improving older populations’ well-being. AI: artificial intelligence; mHealth: mobile health.

The evolution map (Figure 16) traces the thematic development of AI research aimed at improving older people’s well-being across 4 distinct periods. During the foundational stage (2018-2020), the field was rooted in core technological and health care domains, with early emphasis on AI, DH, and mobile and electronic health platforms (eHealth and mHealth). This phase laid the technological groundwork for future advancements. In the subsequent period (2021-2022), the research landscape diversified significantly, incorporating aging, assistive technology, dementia care, and digital literacy. Themes such as machine learning and heart failure management gained momentum, alongside the emergence of older people as a distinct focus group. The 2023-2024 period marks a phase of maturation, characterized by more applied and integrated research. Notable themes included health monitoring technologies (eg, chatbots), responses to COVID-19, depression screening, and the development of user-focused tools like mobile communication and natural language processing. Broader areas such as patient education, telemedicine, and public health also became more prominent. Finally, in 2025, the field further specialized, with increasing attention to cancer care, DH literacy, depression management, and advanced technologies like blockchain, the Internet of Things (IoT), and implementation science. This progression illustrates a shift from general, technology-focused research toward more targeted, condition-specific, and ethically grounded applications. The persistence of foundational themes—such as DH—across all periods, combined with the integration of human-centered approaches and cross-disciplinary technologies, reflects the field’s ongoing transformation into a sophisticated, practical, and socially responsive domain addressing the complex needs of older populations.

#### General Population

**Figure 17.**
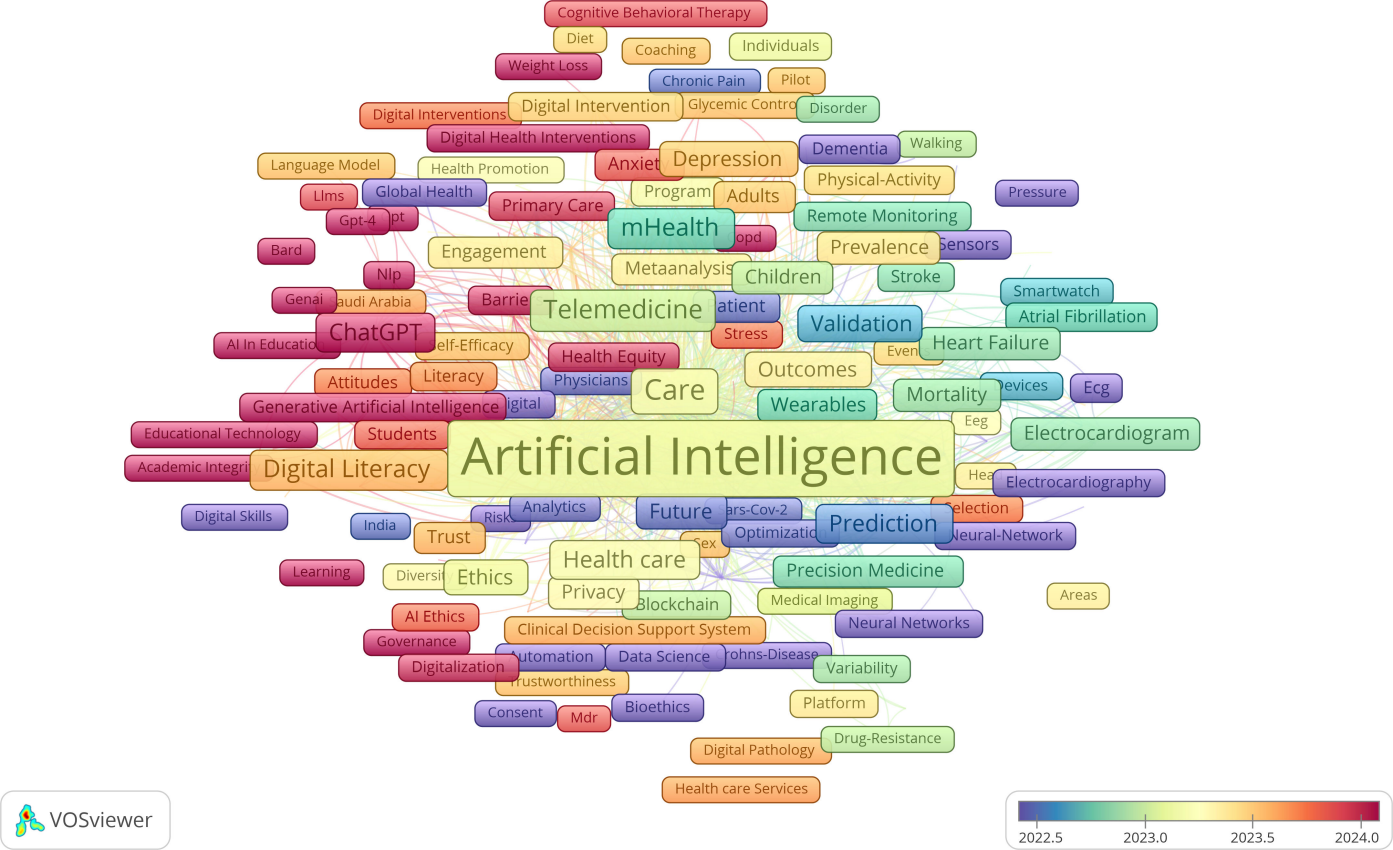
Time overlay network map of the role of artificial intelligence in improving the general population’s well-being.

The overlay map in Figure 17 illustrates the thematic landscape of AI research in older people health care from 2021 to 2025, with AI as the central node connecting a broad range of applications. Surrounding clusters reflect core areas such as DH, telemedicine, chronic disease management, and mental health interventions. Earlier studies (2021-2022) emphasized foundational AI concepts, digital literacy, and dementia care, while recent research (2023-2025) shifted toward personalized medicine, wearable technologies, and predictive analytics. Emerging concerns such as ethics, privacy, and clinical validation gained prominence alongside technologies like chatbots, IoT, and mobile platforms. These developments indicate a maturing field moving from technology acceptance to real-world integration, highlighting AI’s growing role in addressing both medical and psychosocial challenges in aging populations.

**Figure 18.**
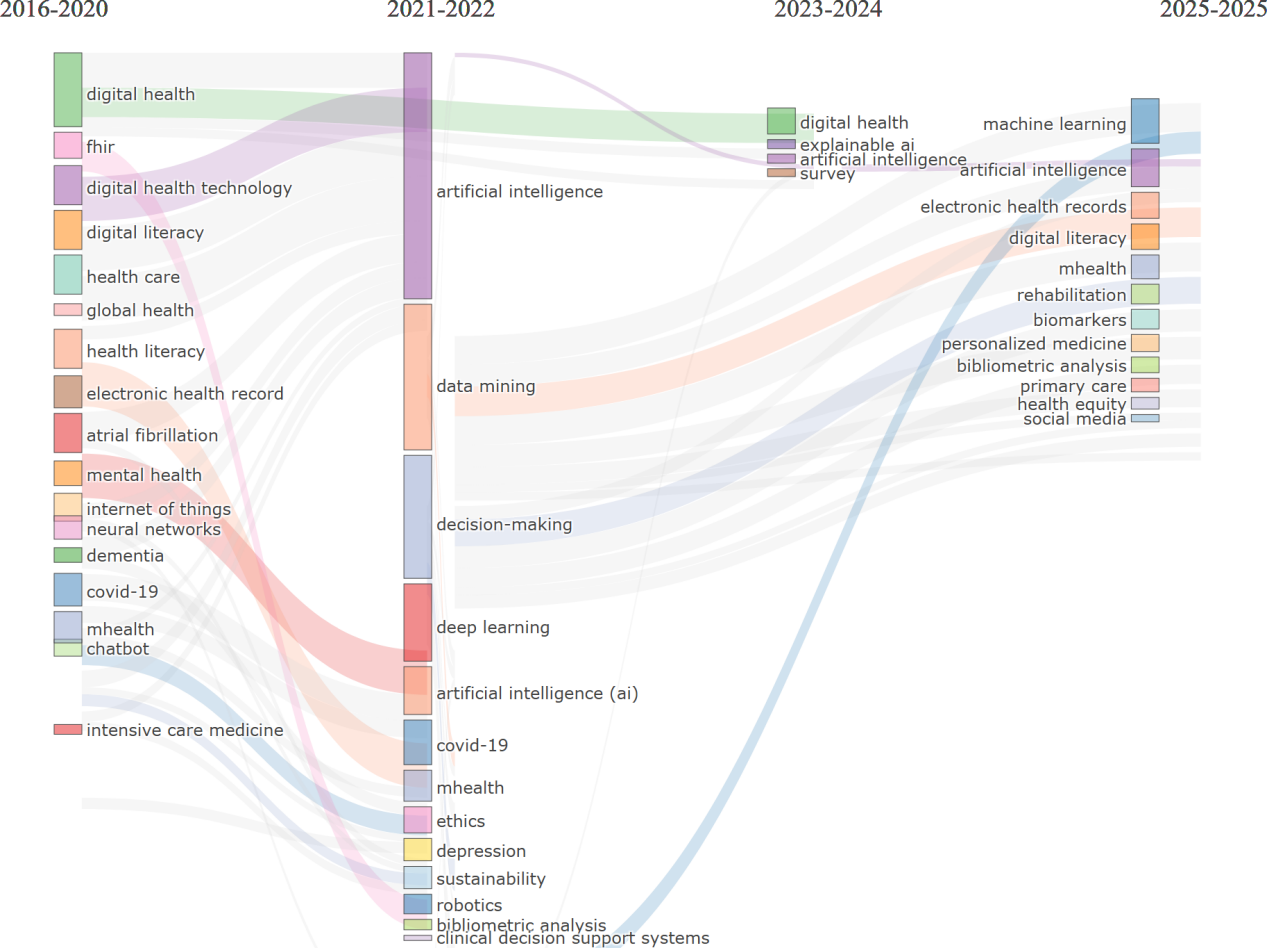
The thematic evolution map for the role of artificial intelligence in improving the general population’s well-being. mHealth: mobile health.

Figure 18 illustrates the evolution of AI research in health care across four periods: (1) 2016‐2020, (2) 2021‐2022, (3) 2023‐2024, and (4) 2025. In the first phase (2016‐2020), research was fragmented, centered on specific health conditions such as dementia and mental health, and marked by early development in digital infrastructure and AI experimentation. From 2021 to 2022, the field shifted significantly, with AI emerging as the central hub, integrating multiple foundational themes—ranging from health care and mental health to neural networks and data mining. This period marked AI’s transition from a supporting tool to a central organizing force, alongside the rise of telemedicine and clinical decision support systems, catalyzed by COVID-19. In 2023‐2024, research consolidated around 3 major themes: DH, AI, and explainable AI. The emergence of explainable AI reflects a critical shift toward trust, interpretability, and human-centered design. This stage also saw more systematic methodologies and validated applications, signaling the field’s maturation. By 2025, research diversified into practical implementation areas such as electronic health records, biostatistics, rehabilitation, and health equity, indicating a movement toward equitable, preventive, and personalized health care. Ethical concerns—privacy, transparency, and access—gained visibility, pointing to a more socially conscious research agenda.

## Discussion

### Principal Findings

The evolution of research on AI applications in digital wellness demonstrates a marked increase in scholarly attention since 2016, with significant growth in both general and older populations. Initially, limited studies focusing on AI’s impact among older adults have expanded considerably, highlighting tailored interventions such as cognitive health monitoring, fall prevention, and chronic disease management. Despite this growth, the research landscape remains fragmented, characterized by small, insular collaborative networks and regional concentration, particularly in older people–focused studies. Influential institutions and authors serve as central nodes within citation networks; yet, cross-group collaboration is limited, potentially restricting interdisciplinary knowledge exchange. Overall, these patterns reflect an emerging but still developing field, underscoring AI’s expanding role in improving digital wellness while revealing the need for more integrated research efforts. This discussion systematically addresses each of the 5 research questions posed in the introduction, providing comprehensive insights into the current state and future directions of this evolving field.

### The Evolution of AI’s Impact on Digital Wellness in the General and Older Populations

The evolution of AI research in digital wellness reveals distinctly different trajectories for the general and older populations, reflecting varying stages of technological adoption and research maturity.

#### Evolution Patterns and Growth Trajectories

AI’s role in improving digital wellness has developed substantially over the past few years. Before 2019, research on the impact of AI in this field was limited, especially among the older population. However, there has been a significant increase in studies and publications on the diversification and impact of AI applications from this year and on. For example, Wilmink et al [28] showed a reduction of 39% in hospitalization and a reduction of 69% in falls among older adults residing in assisted living communities, thanks to the positive impact of AI-driven DH platforms and wearable devices. In addition, Ramesh et al [29] showed that the increase of cloud-based doctor systems supported by recurrent neural networks provides proactive monitoring and personalized care for the management of chronic diseases. Furthermore, extensive DH programs for polychronic conditions have shown positive results, emphasizing the need to integrate different approaches in caring for the complex needs of older adults [30]. This shift highlights a growing recognition of AI’s potential to enhance DH across the general population [2] and among older adults in particular [31,32]. Key themes such as AI in health care, DH, and telemedicine gained prominence during this period [33]. As AI technologies have advanced, they have been increasingly applied in real-world settings to improve health management, assistive technologies, and behavioral interventions [34].

For the general population, AI-driven digital wellness research experienced exponential growth from 2016 onward, with publications remaining below 100 annually until 2022, then surging to 1083 papers in 2024. This dramatic increase demonstrates the field’s rapid maturation and widespread adoption of AI technologies across diverse health care applications. The polynomial curve fitting showed a very high coefficient of determination (*R*²=0.8778), indicating a predictable and sustained growth pattern that suggests continued expansion in the coming years.

In contrast, research focusing on the older population followed a more gradual development path. Initially, there were few publications on AI-driven wellness solutions among the older population, though since 2020, the number of studies has grown, indicating the recognition of the unique challenges the older populations face [35]. This shift emphasizes a growing understanding of the need to devote greater attention to the specific digital wellness needs of older adults through AI-based interventions, such as addressing cognitive decline and dementia; according to Rutkowski et al [36], Graham and Depp [37], and Wong et al [38], cognitive decline and dementia are conditions that are expected to affect 150 million people globally by 2050 [39]. Graham and Depp [37] state that these AI technologies are intended to make it easier to detect cognitive impairments in their early stage and monitor them by using algorithms of machine learning to analyze large datasets to build predictive models and gain insights. Zhang et al [40] and Chien et al [41] support these findings and add that safety monitoring systems, supported by AI technology, are being adopted to improve older people’s quality of life within both their communities and home environments. AI applications targeting dementia care, risk management, and mHealth emerged in publications after 2020 [42]. The analysis has shown that based on the fitting curve (as shown in Figure 2), we can predict that in the upcoming years, the annual volume of publications, for both the general and older population, will continue to grow in the upcoming years. The growing body of research on AI applications for older adults, as evidenced by a sharp increase in publications particularly from 2014 to 2022, reflects rising scholarly and societal interest in improving the quality of life for older individuals through technology [43].

#### Citation Impact and Knowledge Dissemination

Both populations demonstrated similar average citation rates (general: 13.7 vs older people: 13.5), indicating comparable research quality and impact despite volume differences. However, citation patterns revealed different dynamics: general population research peaked in citations during 2023 (10,547 citations) before declining, while older people–focused research showed more consistent citation accumulation, peaking in 2020 (1600 citations) with subsequent stabilization. This suggests that older people–focused research may have a more sustained impact, potentially due to its specialized nature and targeted application domains.

#### Thematic Evolution Over Time

The thematic evolution analysis revealed distinct developmental phases for both populations. General population research progressed from foundational AI concepts (2016‐2020) through integration phases (2021-2022) to specialized applications (2023-2025), with recent emphasis on explainable AI, electronic health records, and health equity. Older people–focused research showed a more targeted evolution, beginning with basic DH concepts (2018-2020), expanding to include aging-specific concerns like dementia care and assistive technology (2021-2022), then advancing to specialized applications including telemedicine, depression screening, and IoT integration (2023‐2025).

### Global Collaboration Patterns in AI Research on Digital Wellness

The analysis revealed distinct collaboration patterns that reflect both the global nature of AI research and the specialized requirements of older people–focused studies.

#### General Population Collaboration Networks

Research on the general population demonstrated extensive global collaboration, with 82 countries meeting the publication threshold and forming four major clusters: (1) a broad alliance led by the United States, China, and the United Kingdom, including various African and Asian nations; (2) a European-Asian cluster centered on Germany; (3) a Canadian-Middle Eastern partnership; and (4) a European consortium led by Italy and Spain. This network structure indicates mature, well-established international research partnerships with strong intercontinental connections.

Key bridging countries—Australia, South Africa, and India—emerged as critical intermediaries, facilitating knowledge exchange between otherwise separate regional clusters. These nations enhance global integration by connecting different research traditions and methodological approaches, contributing to the field’s rapid advancement and diverse application contexts.

#### Older Population Collaboration Patterns

Older people–focused research revealed a more concentrated collaboration network, with 34 countries participating and forming 3 primary clusters. The United States maintained its central role, while strong regional partnerships emerged between European countries and between Asian nations. Canada, Brazil, Finland, and Israel served as bridge countries, facilitating cross-regional collaboration despite the overall more limited scope compared to general population research.

This more constrained network structure reflects several factors: the specialized nature of older people–focused research, potentially higher barriers to international coordination for vulnerable population studies, and the field’s relative immaturity. However, the quality of collaborations appears strong within established clusters, suggesting effective regional partnerships that could serve as foundations for broader international expansion.

Research on AI in the domain of digital wellness, specifically in relation to the older population, is characterized by a complex web of international collaboration, albeit with a more fragmented landscape when compared to the general population. For the general population, the United States, the United Kingdom, China, and other Western nations dominate the research landscape, forming 2 primary collaboration clusters centered around North America and Europe and the other around China and the Middle East. Turkey and Russia serve as bridging points between these 2 clusters. This broader, global research network is represented by a large number of sources, which collectively contributed to a significant body of papers.

In contrast, the older population’s research network is relatively more concentrated in specific regions such as North America, Europe, and parts of Asia, with the United States being the dominant hub. This smaller, more regionally focused collaboration network reflects the emerging, but still underdeveloped, nature of research on AI for older adults.

### Leading Institutions in AI Research for Digital Wellness

Institutional leadership patterns reveal both convergent and divergent trends between general and older population research, with implications for research capacity and future development.

#### Leading Institutions and Research Capacity

For general population research, 3143 institutions participated, with top contributors including University of London (365/3143, 11.6%), Harvard University (316/3143, 10.1%), University of Toronto (281/3143, 8.9%), Mayo Clinic (219/3143, 7%), and University College London (219/3143, 7%). The extensive institutional involvement (900 institutions publishing 3 or more papers, with 880 actively collaborating) demonstrates the field’s broad appeal and interdisciplinary nature across medical, technological, and social science domains.

Older people–focused research involved 757 institutions, led by University of Toronto (77/757, 10.2%), University of London (69/757, 9.1%), National University of Singapore (48/757, 6.3%), University College London (48/757, 6.3%), and Harvard University (43/757, 5.7%). While the absolute numbers are smaller, the concentration of high-quality research among leading institutions suggests strong specialized expertise development.

Institutions contributing to AI and digital wellness research also reflect these patterns. Top institutions including Harvard University, Mayo Clinic, and University of Toronto have been pivotal in advancing research on AI for the general population, whereas institutions such as the University of Toronto, University College London, and Mayo Clinic have played a central role in older people–focused research. However, the number of institutions contributing to research on older people is far smaller, as is the number of institutions forming collaborative networks. This suggests that while AI’s role in digital wellness is a recognized field, the research on its different applications for older people is still in its infancy and is more regionally concentrated.

#### Institutional Collaboration Patterns

The institutional collaboration networks mirror the country-level patterns, with general population research showing extensive interinstitutional partnerships across 880 collaborating institutions, while older people research demonstrated more focused collaboration among 62 institutions. This difference suggests that older people–focused research may benefit from more targeted funding and collaboration initiatives to expand institutional participation and cross-institutional knowledge sharing.

#### Regional and Disciplinary Distribution

Leading institutions span multiple continents and disciplinary backgrounds, from medical schools (Harvard and Mayo Clinic) to comprehensive universities (University of Toronto and University of London) and specialized technological institutes. This diversity indicates the field’s inherently interdisciplinary nature and the need for continued cross-sectoral collaboration to address the complex challenges of AI-driven digital wellness.

The findings reveal a clear pattern of limited collaboration and fragmentation within the research community studying AI applications for health and well-being. Despite a growing body of work in both older people–focused and general population research, relatively few researchers contribute multiple publications, and even fewer appear to engage in sustained collaborative efforts. Coauthorship networks tend to be small and internally cohesive, with minimal interaction across research groups. This suggests that the field is largely driven by isolated teams rather than integrated, interdisciplinary communities.

Such fragmentation may reflect the early or exploratory nature of the field, where researchers operate within specific institutional or disciplinary boundaries. However, this lack of cross-group collaboration could hinder the development of more holistic, impactful solutions, particularly in a domain that relies on the convergence of technology, medicine, behavioral science, and policy. The absence of broader cooperation may also limit knowledge transfer and the sharing of best practices across contexts and populations.

### Key Journals and Publication Trends in This Field

Publication patterns reveal the field’s evolving scholarly infrastructure and the emergence of specialized venues for older people–focused research.

#### Journal Landscape and Publication Venues

General population research spans 1171 journals, with top publications including *Digital Health* (331/3429, 9.7%), *Frontiers in Digital Health* (250/3429, 7.3%), *Journal of Medical Internet Research* (185/3429, 5.4%), *PLOS Digital Health* (151/3429, 4.4%), and *Lancet Digital Health* (143/3429, 4.2%). This broad journal distribution indicates the field’s interdisciplinary nature and integration across medical informatics, health care services, computer science, and public health domains.

Older people–focused research concentrated in 147 journals, led by *The Lancet Digital Health* (33/344, 9.6%), *Digital Health* (29/344, 8.4%), *Journal of Medical Internet Research* (24/344, 7%), and several specialized venues, each contributing 14 publications. The higher concentration in fewer journals suggests a more specialized publication ecosystem that may benefit from expansion to increase visibility and accessibility.

#### Research Areas and Categories

Both populations showed similar emphasis on medical informatics (general: 1411/3411, 41.4% vs older people: 163/344, 47.4%) and health care science services (general: 1357/3411, 39.8% vs older people: 129/344, 37.5%), indicating shared foundational interests. However, notable differences emerged in specialized areas: older people research placed greater emphasis on geriatrics and gerontology (older people: 32/344, 9.3% vs minimal in the general population: 8/3411, 0.2%) and maintained a strong focus on public health applications (older people: 58/344, 16.9% vs general: 506/3411, 14.8%).

When analyzing scientific publications for both the general and older populations, notable differences emerged in research scope and publication volume. Research on the general population included 3429 papers across 1171 journals, with top outlets such as *Digital Health*, *Frontiers in Digital Health*, and *Journal of Medical Internet Research* accounting for a substantial share. The leading research areas were medical informatics, health care science services, and computer science, while the most prominent publication categories—according to WoS—were medical informatics, health care science services, and public environmental occupational health, alongside health policy services and general internal medicine.

In contrast, older people–focused literature comprised 344 papers published in 147 journals, with *The Lancet Digital Health* and *Digital Health* emerging as the most frequent sources. Despite the lower volume, research on older people emphasized similar domains, particularly medical informatics and health care science services. However, it placed relatively more emphasis on public environmental occupational health, geriatrics and gerontology, and general internal medicine. The category distribution further highlighted the relevance of health policy services for aging populations, suggesting a research shift toward addressing the specific health and policy needs of older adults.

#### Publication Quality and Impact

The presence of high-impact journals like *The Lancet Digital Health* and established venues like *Journal of Medical Internet Research* in both publication lists indicates strong research quality across populations. However, the emergence of newer, specialized journals (*Digital Health* and *Frontiers in Digital Health*) suggests the field’s rapid evolution and the need for diverse publication venues to accommodate different research approaches and target audiences.

### Emerging Themes in AI Research for Digital Wellness

Keyword analysis and thematic evolution mapping revealed distinct research themes and emerging trends that reflect both technological advancement and population-specific needs.

#### General Population Research Themes

The general population research encompassed 11,473 keywords, with dominant themes including AI (n=1678), DH (n=990), machine learning (n=436), care (n=224), and deep learning (n=189). Six major thematic clusters emerged, representing (1) core AI technologies and machine learning applications, (2) DH infrastructure and telemedicine, (3) chronic disease management and clinical applications, (4) mental health and behavioral interventions, (5) data science and predictive analytics, and (6) ethical considerations and implementation challenges.

Recent thematic developments (2023‐2025) emphasize explainable AI, personalized health care, electronic health record integration, and health equity considerations. These trends indicate the field’s maturation from basic AI applications toward more sophisticated, ethically informed, and socially responsible implementations. The thematic evolution maps (Figures17  18) show that between 2016 and 2020, keywords were more condition-specific (eg, “dementia” and “mental health”) and focused on foundational technologies (“neural networks” and “digital health”). From 2021 to 2022, the field consolidated around “artificial intelligence” as a central hub, integrating emerging themes such as “deep learning,” “data mining,” and “telemedicine.” In 2023‐2024, we see a strong emergence of “explainable AI” and “digital health” as interconnected cores, reflecting a shift toward trust, usability, and integration into clinical contexts. By 2025, new themes like “health equity,” “personalized medicine,” and “rehabilitation” appear, suggesting a broadening of AI applications toward socially conscious and condition-specific interventions.

The practical applications of AI in digital wellness are diverse and continually developing for both populations. Among the general population, AI is increasingly embedded into mHealth apps, telemedicine, and wearables, with a growing focus on personalized health care and disease prevention. Notable trends include the use of AI to manage chronic conditions such as cardiovascular diseases, diabetes, and mental health conditions. The application of AI to monitor physical activity, detect early warning signs of disease, and support clinical decision-making has proven to be particularly beneficial, as shown by the exponential increase in publications post-2019, reaching 717 papers in 2024 for the general population. This growth trajectory is reflected in the high citation impact of these papers, with an average citation rate of 12.6 per paper and a peak of 6080 total citations in 2021.

#### Older Population Research Themes

Older people–focused research involved 2102 keywords, with key themes including AI (n=154), DH (n=126), machine learning (n=37), mHealth (n=24), care (n=24), and dementia (n=22). Four primary clusters emerged: (1) AI technologies adapted for aging populations, (2) mHealth and assistive technologies, (3) dementia care and cognitive health monitoring, and (4) risk management and chronic condition support.

The thematic evolution for older populations showed progression from basic aging concepts (2018‐2020) through technology integration (2021‐2022) to specialized applications including telemedicine, social isolation interventions, and IoT-based monitoring systems (2023‐2025). Notably, themes like “social isolation,” “fall detection,” and “digital literacy” became increasingly prominent, reflecting the field’s growing attention to older people–specific challenges. The older people thematic evolution maps (Figures15  16) illustrate a similar centralization of “artificial intelligence” in 2021‐2022, but with stronger connections to socially relevant and accessibility-focused terms. Early-stage keywords such as “digital literacy,” “dementia,” and “mHealth” persisted across later periods, indicating sustained relevance. From 2023 onward, specialized topics like “social isolation” and “IoT” emerged alongside “telemedicine,” marking a shift toward holistic digital wellness interventions that address both medical and psychosocial needs. By 2025, novel themes like “biomarkers” and “rehabilitation” point to a growing integration of AI into precision health monitoring and recovery processes tailored for older adults.

The development of AI applications that are intended for older people mainly focuses on tailored interventions in fields such as mobility, cognitive wellness, and chronic health management. Studies [44-49] have shown that applications such as virtual health care, fall prevention, and dementia care are gaining more and more momentum, which emphasizes the importance of building AI-driven systems focused on remote monitoring, enhancing the older population’s daily living routines, and reducing their social isolation. Recent work by Makmee and Wongupparaj [50] further supports this direction, demonstrating the effectiveness of virtual reality (VR)–based cognitive interventions—complemented by behavioral and electroencephalography evidence—in improving cognitive functions and well-being among older adults with mild cognitive impairment. AI’s role in assisting aging individuals with activities of daily living, enhancing safety, and improving mental health through virtual assistants or therapy applications represents a promising frontier for improving digital wellness among older people. Smart home systems and wearables have been shown to support independence by providing personalized care, fall detection, and real-time health monitoring [51-55]. These technologies not only enhance safety and autonomy but also address barriers such as usability, cost, and privacy while offering opportunities for improved health outcomes and quality of life.

#### Emerging Interdisciplinary Themes

Several cross-cutting themes emerged across both populations: (1) ethical AI implementation, with growing emphasis on transparency, fairness, and accountability; (2) personalized health care approaches leveraging AI for individualized interventions; (3) integration of IoT and wearable technologies for continuous monitoring; (4) social and behavioral factors in technology adoption; and (5) policy and implementation science considerations for real-world deployment.

For older populations specifically, emerging themes include (1) age-friendly AI design principles, (2) intergenerational technology support models, (3) cognitive accessibility in AI interfaces, (4) privacy and security considerations for vulnerable populations, and (5) family and caregiver integration in AI-supported care systems.

The rapid growth of publications in this field after 2019 corresponds with evolving research themes identified in keyword co-occurrence and topic modeling analyses. Key terms such as personalized health care, chronic disease management, virtual assistants, and smart home systems have emerged as dominant themes. For the older people subgroup, keywords highlight a growing interest in fall detection, dementia care, social isolation, and ethical AI. These patterns suggest a shifting research focus from general DH applications toward more inclusive and population-specific interventions, reinforcing the importance of designing AI solutions that address both medical and psychosocial aspects of well-being.

### Synthesis and Future Directions

As the global population continues to age, there is a need to strengthen international and institutional collaborations in developing models and AI technologies dedicated to improving older people’s digital well-being. Given the rapidly increasing research interest in AI applications for older adults, as evidenced by the exponential growth in scientific publications, expanded interdisciplinary and international collaborations are essential to address the growing demand for digital solutions.

Future research directions should address several critical gaps identified in the current literature. First, the acceptance of AI-based conversational agents for managing noncommunicable diseases among older adults remains inadequately evaluated, presenting a significant opportunity for research [8]. Studies should focus on adapting established acceptance frameworks to specific health care contexts and emerging AI technology innovations, particularly, as AI chatbots and virtual health assistants become more prevalent in health care delivery.

Future studies should strive to clarify the impact and effectiveness of tailored AI interventions for older people, testing their therapeutic effectiveness, ethical implications, accessibility, and socioeconomic influence [56]. The current bibliometric analysis revealed a discontinuous research network between countries regarding research focused on older people, suggesting, as noted by Koç [57], an opportunity for extensive global knowledge-sharing and research collaborations.

Research should also investigate the role of visual demonstrations in enhancing technology acceptance, as a meta-analysis made by Yang et al [10] suggests that visual demonstrations significantly enhance both perceived usefulness and social influence relationships with behavioral intention. This finding has practical implications for technology training programs and interface design for older adults. Future research should explore the underlying cultural, economic, and infrastructural factors that contribute to regional differences in technology acceptance among older adults. Such studies could inform culturally sensitive technology design and implementation strategies.

The accessibility and use of AI within wellness systems among the older population should be given adequate attention, as this is a subgroup that is often challenged by the use of technology. Following the findings of Htet et al [58] and Zhao and Li [59], policymakers, health care providers, and technology developers must work together to ensure that AI tools are designed in a way that is inclusive and user-friendly for older individuals, enabling them to harness the full potential of digital wellness innovations. Specifically, Li et al [60] and Wu et al [61] suggested that a person-centered approach should be prioritized to ensure that these AI-driven systems are equitable, transparent, and validated for the older population. This approach should include innovations such as AI-driven VR games and smart older people care systems, provide real-time support, and enhance social connectivity and well-being.

Longitudinal studies examining the transition from acceptance to sustained use are crucial, particularly focusing on continuance intention and long-term adherence to DH technologies [62]. Research should investigate how initial acceptance factors evolve over time and identify critical points where interventions might be most effective in maintaining engagement.

The development and validation of age-specific TAMs is another important research direction. While TAM and the unified theory of acceptance and use of technology provide valuable frameworks, studies suggest that additional constructs such as perceived irreplaceability, perceived credibility, and compatibility may be particularly relevant for older adults [62]. Future research should work toward developing comprehensive models that better capture the unique considerations of older technology users.

Finally, research should focus on preparing middle-aged adults for aging through technological competency development [16]. This proactive approach could help address the digital divide before it becomes entrenched, with studies needed to identify optimal timing, methods, and content for technology preparation interventions targeting pre-older populations.

In addition, future research should provide more specific methodological guidance to advance the field. For example, mixed methods designs combining quantitative longitudinal data with qualitative insights from older people can elucidate both adoption patterns and lived experiences with AI technologies. Experimental studies testing the efficacy of tailored AI interventions, such as AI-driven virtual assistants or VR rehabilitation tools, would clarify therapeutic benefits and user engagement. Methodological innovations like ecological momentary assessment and real-time data capture through wearables can provide granular insights into daily technology use and health outcomes. Interdisciplinary collaborations should be encouraged between computer scientists, gerontologists, behavioral economists, ethicists, and health care practitioners to foster holistic AI solutions that are technically sound, ethically responsible, and aligned with older adults’ needs. Platforms enabling open data sharing and multisite trials would accelerate knowledge accumulation and generalizability. Addressing ethical considerations, such as transparency, privacy, and consent, should be embedded throughout the research design. These focused recommendations aim to guide researchers in designing rigorous, relevant, and impactful studies that move beyond acceptance to sustained, equitable AI adoption among aging populations.

Finally, the future of AI in digital wellness holds immense promise. Still, as also mentioned by Zhao and Li [59] and Eziamaka et al [63], it requires continued research, collaboration, and thoughtful implementation to ensure that it benefits all demographic groups, especially older people, in a way that is equitable, effective, and sustainable.

The systematic analysis of this study’s 5 research questions reveals both the promising trajectory of AI in digital wellness and the critical need for more inclusive, older people–focused research and development. While general population research has achieved significant scale and international collaboration, older people–focused research remains more specialized and regionally concentrated, representing both a challenge and an opportunity for field development.

The convergence of themes around personalized care, ethical implementation, and real-world application suggests the field’s evolution toward more mature, socially responsible AI deployment. However, the persistent gaps between general and older population research indicate the need for targeted interventions to ensure equitable technological advancement and accessibility.

Future research should prioritize (1) expanding international collaboration in older people–focused AI research; (2) developing age-appropriate AI technologies that address specific challenges of aging populations; (3) strengthening the publication infrastructure for older people–focused digital wellness research; (4) fostering interdisciplinary partnerships that bridge technological innovation with gerontological expertise; and (5) ensuring ethical, accessible, and culturally sensitive AI implementations that serve diverse aging populations worldwide.

### Policy Implications and Recommendations

Building on the evidence presented in this study, several targeted policy interventions are necessary to bridge the existing research and implementation gaps in AI applications for older people’s digital wellness. National research funding agencies, such as the National Institutes of Health, UK Research and Innovation, and Horizon Europe, should explicitly prioritize funding for AI projects that focus on aging populations. Our findings indicate a disproportionately smaller volume of older people–specific research despite global demographic trends. To address this, funding calls should require the inclusion of older adults as a central population group; promote interdisciplinary research designs involving gerontology, computer science, and public health; and mandate ethical assessments tailored to this demographic.

In addition to strategic funding, there is a clear need to incentivize cross-sector collaboration. Policymakers should support or cosponsor research initiatives that foster partnerships among universities, health care providers, and technology developers. The fragmented nature of coauthorship and institutional networks in older people–focused AI research underscores the value of consortia-based models. Initiatives similar to the European Union’s Horizon AI and Aging programs could encourage data sharing, coordinated trials, and the scaling of successful tools.

To ensure inclusivity in technological design, regulatory bodies must implement guidelines that mandate age-friendly design principles in DH technologies. These should encompass enhanced accessibility features such as larger interfaces and audio guidance, digital literacy support, and simplified user interactions. Existing frameworks, like the Web Content Accessibility Guidelines, can serve as a model for integrating older people’s usability criteria into national and international AI standards.

Public health policy should also prioritize the development of community-based digital literacy programs. Lifelong learning initiatives, especially at the municipal and regional levels, can play a vital role in empowering older adults. AI-supported tools—such as virtual tutors or gamified interfaces—should be leveraged to build digital confidence and self-efficacy among older people. Partnerships with nongovernmental organizations, older people centers, and libraries can facilitate the implementation of “AI Literacy for Seniors” programs that align with broader technological transformations in health care and public services.

Furthermore, ethical oversight of AI systems must be enhanced through the inclusion of gerontological expertise on ethics review boards. This would help ensure that reviews account for the unique vulnerabilities of aging populations, including algorithmic bias, informed consent challenges, and data sensitivity. International bodies such as the World Health Organization, Organisation for Economic Co-Operation and Development, and national AI task forces can integrate this recommendation into their broader AI governance structures.

Finally, the establishment of robust monitoring and evaluation frameworks is essential to assess the impact of AI interventions on older people’s wellness. These systems should track clinical outcomes as well as psychosocial well-being, levels of digital engagement, and barriers to technology adoption. The development of standardized indicators and public reporting mechanisms will support transparency, informed resource allocation, and evidence-based policymaking.

These policy recommendations align with global priorities, including the World Health Organization’s “Decade of Healthy Ageing” and the United Nations Sustainable Development Goals (SDG; specifically SDG 3 and SDG 10). By embedding these strategies into national and international policy agendas, stakeholders can ensure that AI-driven digital wellness initiatives contribute to more equitable health outcomes and do not inadvertently exacerbate existing disparities among older adults.

### Limitations and Potential Biases

This study has several limitations that should be acknowledged. First, the analysis relied exclusively on the WoS Core Collection, which, although known for its high-quality indexing, may exclude relevant studies found in other databases such as Scopus, PubMed, or regional and domain-specific repositories, thus introducing database bias. Second, only English-language publications were included, potentially omitting significant contributions published in other languages (language bias). Third, the analysis focused on peer-reviewed papers, reviews, and conference proceedings, thereby excluding gray literature, policy reports, and other nonindexed formats that may contain valuable insights (publication bias). Citation bias is also a concern, as bibliometric visualizations often emphasize highly cited papers, favoring older or more mainstream studies while underrepresenting newer or niche research. Temporal bias may have occurred, particularly for publications from 2024 to 2025, which may not yet have had sufficient time to accumulate citations. Keyword selection bias may have affected retrieval, as the search strategy was limited to predefined terms related to AI, digital wellness, and aging; relevant papers using alternative or emerging terminology may have been excluded. Additionally, visualization tools such as VOSviewer and Bibliometrix, while robust, are influenced by algorithmic thresholds and clustering techniques that can oversimplify complex thematic structures. The binary logic used in the SRCH_STR_ALL and SRCH_STR_OLD search strings may have artificially separated studies that address both general and older populations. Finally, the interpretation of bibliometric maps and clusters involves an element of subjectivity, as visual proximity does not always reflect substantive thematic or intellectual similarity.

### Conclusions

This study provided an in-depth bibliometric analysis of the intersection between AI and digital wellness with a comparison of the older population to the general population. The findings demonstrate a rapid growth in AI research across both the general and older populations, highlighting key trends, challenges, and opportunities within the field. Although AI-driven digital wellness has garnered increasing attention in recent years, it is evident that there remain substantial gaps, particularly in addressing the unique needs of older adults.

Key findings from our analysis reveal markedly different development trajectories between general and older people–focused research in AI-driven digital wellness. General population studies expanded rapidly over recent years, while older people–focused research followed a slower, more gradual path, gaining momentum only in the past few years. Although both areas achieved comparable citation impact, older people research demonstrated more consistent long-term influence. Collaboration patterns also differed, with general studies forming extensive, globally connected networks, whereas older people research remained concentrated in fewer countries and clusters, indicating untapped opportunities for international partnerships. Institutional participation was far broader in general research, while older people–focused studies were driven by a smaller set of leading organizations. Publication patterns reflected these differences, with older people research concentrated in a limited number of journals emphasizing geriatrics, gerontology, and public health, compared to the broader medical informatics scope of general studies. Thematic analysis showed general research advancing toward explainable AI and health equity, while older people–focused work prioritized dementia care, assistive technologies, and IoT-based monitoring, alongside emerging attention to issues such as social isolation and digital literacy. However, limited cross-population collaboration and network fragmentation remain as barriers to integrated, holistic solutions. Addressing these gaps will require targeted policy measures, including age-friendly design standards, digital literacy programs, dedicated funding mechanisms, and strengthened ethical oversight for AI serving older populations.

In conclusion, this research contributes valuable insights into the role of AI in enhancing the digital wellness of older people while also highlighting the disparities between the general and older populations in terms of research focus and technological adoption. As the field continues to evolve, it is crucial that future policy, research, and development efforts prioritize the inclusion of vulnerable populations. By addressing the unique needs of older adults, society can ensure that they are not only able to keep pace with technological advancements but are also empowered to thrive in an increasingly AI-driven world.

Looking toward the future, AI research in improving digital well-being among older people holds immense promise for transforming how we approach aging in the digital era. The convergence of emerging technologies—including explainable AI, IoT, VR, and advanced machine learning algorithms—presents unprecedented opportunities to develop more personalized, accessible, and effective interventions for older populations. Future research is likely to focus on creating AI systems that not only address medical and physical health needs but also tackle psychosocial challenges such as social isolation, depression, and cognitive decline through intelligent companion systems, predictive health monitoring, and adaptive user interfaces.

The evolution toward more human-centered AI design will be particularly crucial, emphasizing transparency, trust-building, and ethical considerations that are paramount when serving vulnerable populations. As our analysis demonstrates, the field is moving from basic technology acceptance toward sophisticated, real-world implementations that integrate seamlessly into older adults’ daily lives. Future developments will likely prioritize cultural sensitivity, intergenerational connectivity, and the creation of AI ecosystems that empower rather than replace human agency.

Furthermore, the anticipated expansion of international collaboration networks and the establishment of standardized evaluation frameworks will accelerate the translation of research findings into practical, scalable solutions. The next decade will likely witness the emergence of comprehensive AI-powered platforms that holistically address the complex, interconnected challenges of aging while ensuring digital equity and inclusion. Success in this endeavor will require continued interdisciplinary collaboration, sustained investment in older people–focused research, and a commitment to developing technologies that truly serve the diverse needs and preferences of aging populations worldwide.

## Supplementary material

10.2196/71248 Multimedia Appendix 1 Classification of paper categories for the general population.

## Abbreviations

AI: artificial intelligence
DH: digital health
IoT: Internet of Things
mHealth: mobile health
SDG: Sustainable Development Goal
TAM: technology acceptance model
VR: virtual reality
WoS: Web of Science
