# Supplementary material for: The Role of AI in Improving Digital Wellness Among Older Adults: Comparative Bibliometric Analysis
Source: JMIR AI. 2026 Jan 14;5:e71248. doi: 10.2196/71248 (PMC12808873; doi:10.2196/71248)
Supplement: Multimedia Appendix 1 [file ai-v5-e71248-s001.docx]

| **Web of Science categories** | **# of papers** | **% of total papers** |
| --- | --- | --- |
| Medical Informatics | 1411 | 41.37% |
| Health Care Sciences Services | 1339 | 39.26% |
| Public Environmental Occupational Health | 506 | 14.83% |
| Health Policy Services | 401 | 11.76% |
| Medicine General Internal | 298 | 8.74% |
| Computer Science Interdisciplinary Applications | 288 | 8.44% |
| Cardiac Cardiovascular Systems | 225 | 6.60% |
| Computer Science Artificial Intelligence | 205 | 6.01% |
| Computer Science Information Systems | 183 | 5.37% |
| Engineering Biomedical | 143 | 4.19% |
| Education Educational Research | 136 | 3.99% |
| Computer Science Theory Methods | 132 | 3.87% |
| Engineering Electrical Electronic | 88 | 2.58% |
| Education Scientific Disciplines | 57 | 1.67% |
| Multidisciplinary Sciences | 53 | 1.55% |
| Pharmacology Pharmacy | 48 | 1.41% |
| Psychiatry | 47 | 1.38% |
| Telecommunications | 45 | 1.32% |
| Computer Science Cybernetics | 44 | 1.29% |
| Oncology | 40 | 1.17% |
| Information Science Library Science | 37 | 1.09% |
| Environmental Sciences | 36 | 1.06% |
| Surgery | 35 | 1.03% |
| Engineering Multidisciplinary | 34 | 1.00% |
| Instruments Instrumentation | 34 | 1.00% |
| Chemistry Analytical | 33 | 0.97% |
| Endocrinology Metabolism | 32 | 0.94% |
| Nursing | 31 | 0.91% |
| Computer Science Software Engineering | 30 | 0.88% |
| Biotechnology Applied Microbiology | 29 | 0.85% |
| Neurosciences | 29 | 0.85% |
| Radiology Nuclear Medicine Medical Imaging | 29 | 0.85% |
| Medicine Research Experimental | 28 | 0.82% |
| Green Sustainable Science Technology | 27 | 0.79% |
| Clinical Neurology | 26 | 0.76% |
| Communication | 26 | 0.76% |
| Environmental Studies | 23 | 0.67% |
| Social Sciences Interdisciplinary | 23 | 0.67% |
| Pediatrics | 22 | 0.65% |
| Physics Applied | 21 | 0.62% |
| Management | 20 | 0.59% |
| Genetics Heredity | 18 | 0.53% |
| Gastroenterology Hepatology | 16 | 0.47% |
| Mathematical Computational Biology | 16 | 0.47% |
| Business | 15 | 0.44% |
| Chemistry Multidisciplinary | 15 | 0.44% |
| Infectious Diseases | 15 | 0.44% |
| Materials Science Multidisciplinary | 15 | 0.44% |
| Rheumatology | 15 | 0.44% |
| Psychology Multidisciplinary | 14 | 0.41% |
| Ergonomics | 13 | 0.38% |
| Biochemistry Molecular Biology | 12 | 0.35% |
| Linguistics | 11 | 0.32% |
| Rehabilitation | 11 | 0.32% |
| Cell Biology | 10 | 0.29% |
| Economics | 10 | 0.29% |
| Peripheral Vascular Disease | 10 | 0.29% |
| Robotics | 10 | 0.29% |
| Computer Science Hardware Architecture | 9 | 0.26% |
| Dentistry Oral Surgery Medicine | 9 | 0.26% |
| Ethics | 9 | 0.26% |
| Law | 9 | 0.26% |
| Medical Ethics | 9 | 0.26% |
| Medical Laboratory Technology | 9 | 0.26% |
| Operations Research Management Science | 9 | 0.26% |
| Ophthalmology | 9 | 0.26% |
| Otorhinolaryngology | 9 | 0.26% |
| Primary Health Care | 9 | 0.26% |
| Psychology Clinical | 9 | 0.26% |
| Urology Nephrology | 9 | 0.26% |
| Biophysics | 8 | 0.24% |
| Engineering Industrial | 8 | 0.24% |
| Geriatrics Gerontology | 8 | 0.24% |
| Humanities Multidisciplinary | 8 | 0.24% |
| Mathematics Applied | 8 | 0.24% |
| Nanoscience Nanotechnology | 8 | 0.24% |
| Psychology Experimental | 8 | 0.24% |
| Social Sciences Biomedical | 8 | 0.24% |
| Sociology | 8 | 0.24% |
| Immunology | 7 | 0.21% |
| Language Linguistics | 7 | 0.21% |
| Orthopedics | 7 | 0.21% |
| Sport Sciences | 7 | 0.21% |
| Physiology | 6 | 0.18% |
| Social Issues | 6 | 0.18% |
| Allergy | 5 | 0.15% |
| Automation Control Systems | 5 | 0.15% |
| Biochemical Research Methods | 5 | 0.15% |
| Dermatology | 5 | 0.15% |
| Food Science Technology | 5 | 0.15% |
| Hospitality Leisure Sport Tourism | 5 | 0.15% |
| Imaging Science Photographic Technology | 5 | 0.15% |
| Nutrition Dietetics | 5 | 0.15% |
| Obstetrics Gynecology | 5 | 0.15% |
| Psychology Developmental | 5 | 0.15% |
| Public Administration | 5 | 0.15% |
| Regional Urban Planning | 5 | 0.15% |
| Respiratory System | 5 | 0.15% |
| Statistics Probability | 5 | 0.15% |
| Tropical Medicine | 5 | 0.15% |
| Agriculture Multidisciplinary | 4 | 0.12% |
| Biology | 4 | 0.12% |
| Business Finance | 4 | 0.12% |
| Critical Care Medicine | 4 | 0.12% |
| Parasitology | 4 | 0.12% |
| Psychology Biological | 4 | 0.12% |
| Acoustics | 3 | 0.09% |
| Energy Fuels | 3 | 0.09% |
| Gerontology | 3 | 0.09% |
| Hematology | 3 | 0.09% |
| Mathematics Interdisciplinary Applications | 3 | 0.09% |
| Microbiology | 3 | 0.09% |
| Political Science | 3 | 0.09% |
| Toxicology | 3 | 0.09% |
| Transplantation | 3 | 0.09% |
| Agronomy | 2 | 0.06% |
| Anesthesiology | 2 | 0.06% |
| Behavioral Sciences | 2 | 0.06% |
| Chemistry Medicinal | 2 | 0.06% |
| Chemistry Physical | 2 | 0.06% |
| Construction Building Technology | 2 | 0.06% |
| Cultural Studies | 2 | 0.06% |
| Development Studies | 2 | 0.06% |
| Electrochemistry | 2 | 0.06% |
| Engineering Civil | 2 | 0.06% |
| Engineering Manufacturing | 2 | 0.06% |
| History Philosophy Of Science | 2 | 0.06% |
| Industrial Relations Labor | 2 | 0.06% |
| International Relations | 2 | 0.06% |
| Materials Science Biomaterials | 2 | 0.06% |
| Medicine Legal | 2 | 0.06% |
| Optics | 2 | 0.06% |
| Philosophy | 2 | 0.06% |
| Physics Mathematical | 2 | 0.06% |
| Psychology | 2 | 0.06% |
| Psychology Social | 2 | 0.06% |
| Quantum Science Technology | 2 | 0.06% |
| Remote Sensing | 2 | 0.06% |
| Social Work | 2 | 0.06% |
| Agricultural Engineering | 1 | 0.03% |
| Anthropology | 1 | 0.03% |
| Developmental Biology | 1 | 0.03% |
| Education Special | 1 | 0.03% |
| Emergency Medicine | 1 | 0.03% |
| Ethnic Studies | 1 | 0.03% |
| Forestry | 1 | 0.03% |
| History | 1 | 0.03% |
| Mechanics | 1 | 0.03% |
| Metallurgy Metallurgical Engineering | 1 | 0.03% |
| Meteorology Atmospheric Sciences | 1 | 0.03% |
| Mining Mineral Processing | 1 | 0.03% |
| Pathology | 1 | 0.03% |
| Physics Fluids Plasmas | 1 | 0.03% |
| Physics Multidisciplinary | 1 | 0.03% |
| Psychology Applied | 1 | 0.03% |
| Psychology Educational | 1 | 0.03% |
| Substance Abuse | 1 | 0.03% |
| Transportation Science Technology | 1 | 0.03% |
| Urban Studies | 1 | 0.03% |
| Virology | 1 | 0.03% |
| Water Resources | 1 | 0.03% |
